# Metagenomic and single-cell RNA-Seq survey of the *Helicobacter* *pylori*–infected stomach in asymptomatic individuals

**DOI:** 10.1172/jci.insight.161042

**Published:** 2023-02-22

**Authors:** Chiara Sorini, Kumar P. Tripathi, Shengru Wu, Shawn M. Higdon, Jing Wang, Liqin Cheng, Sanghita Banerjee, Annika Reinhardt, Taras Kreslavsky, Anders Thorell, Lars Engstrand, Juan Du, Eduardo J. Villablanca

**Affiliations:** 1 Division of Immunology and Allergy, Department of Medicine Solna, Karolinska Institutet and University Hospital, Stockholm, Sweden.; 2 Center of Molecular Medicine, Stockholm, Sweden.; 3 Department of Microbiology, Tumor and Cell Biology, Centre for Translational Microbiome Research, Karolinska Institutet, Stockholm, Sweden.; 4 Ersta Hospital, Stockholm, Sweden.

**Keywords:** Immunology, Microbiology, Bacterial infections, Cellular immune response

## Abstract

*Helicobacter pylori* colonization of the gastric niche can persist for years in asymptomatic individuals. To deeply characterize the host–microbiota environment in *H*. *pylori*–infected (HPI) stomachs, we collected human gastric tissues and performed metagenomic sequencing, single-cell RNA-Seq (scRNA-Seq), flow cytometry, and fluorescent microscopy. HPI asymptomatic individuals had dramatic changes in the composition of gastric microbiome and immune cells compared with noninfected individuals. Metagenomic analysis uncovered pathway alterations related to metabolism and immune response. scRNA-Seq and flow cytometry data revealed that, in contrast to murine stomachs, ILC2s are virtually absent in the human gastric mucosa, whereas ILC3s are the dominant population. Specifically, proportion of NKp44^+^ ILC3s out of total ILCs were highly increased in the gastric mucosa of asymptomatic HPI individuals, and correlated with the abundance of selected microbial taxa. In addition, CD11c^+^ myeloid cells and activated CD4^+^ T cells and B cells were expanded in HPI individuals. B cells of HPI individuals acquired an activated phenotype and progressed into a highly proliferating germinal-center stage and plasmablast maturation, which correlated with the presence of tertiary lymphoid structures within the gastric lamina propria. Our study provides a comprehensive atlas of the gastric mucosa–associated microbiome and immune cell landscape when comparing asymptomatic HPI and uninfected individuals.

## Graphical abstract

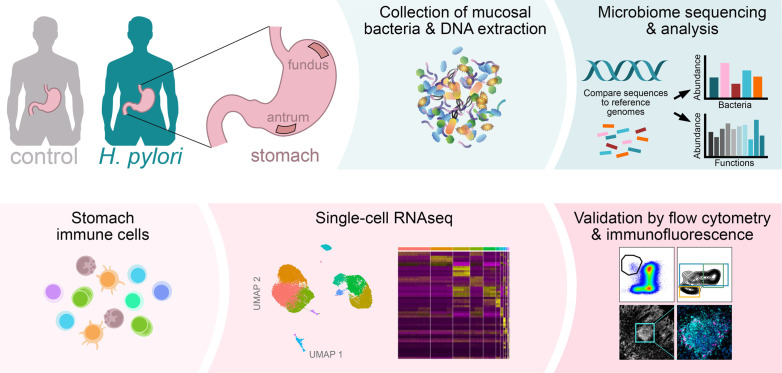

## Introduction

*Helicobacter pylori* chronically infects the gastric epithelium of approximately 50% of the world’s population (1). Symptoms associated with chronic inflammation caused by *H*. *pylori* include loss of appetite, abdominal pain, and unintentional weight loss. Moreover, chronic inflammation induced by *H*. *pylori* is associated with an increased risk of developing severe gastric pathologies, including peptic ulcer and cancer. Next-generation sequencing technology has enabled characterization of the gastric microbiome, which is dominated by the Actinobacteria, Bacteroidetes, Firmicutes, and Proteobacteria in homeostatic conditions (2). The bacterial ecosystem of the gastric niche is significantly affected by *H*. *pylori* infection (3), which may contribute to the broad spectrum of clinical manifestations in chronically infected patients (4–6). However, how *H*. *pylori* infection influences the composition and function of the overall gastric microbiome appears yet to be investigated.

*H. pylori* can prompt gastric epithelial cells to secrete cytokines and chemokines, which eventually recruit innate and adaptive immune cell types (7). Among immune cells or cytokines associated with active protection against infection, IL-17A levels inversely correlated with *H*. *pylori* load in the gastric mucosa, suggesting a role of IL-17–secreting cells in eradicating acute *H*. *pylori* infection (8, 9). However, the presence of Th17 cells has been linked to more severe gastric inflammation and tumor progression in adult patients (10). A recent study of mice with acute *H*. *pylori* infection linked B cell numbers and *H*. *pylori*–specific IgA responses in the gastric mucosa to an expansion of type 2 innate lymphoid cells (ILC2s) (11). The presence of these and other immune cell types in the human stomach, their crosstalk with the human gastric microbiome, and their contributions to human pathology remain topics of debate.

Importantly, 80% to 90% of infected individuals develop long-term infection, and gastritis remains asymptomatic (12). However, whether the changes observed in *H*. *pylori*-infected (HPI) individuals with clinically active gastric disease are also present in those who are asymptomatic is currently unknown. Here, we characterized the gastric microbial microenvironment and immune cell types that accumulate in the gastric mucosa of asymptomatic HPI individuals as compared with uninfected individuals.

## Results

### Composition of the gastric microbiome is severely altered during H. pylori infection.

**Figure 1:**
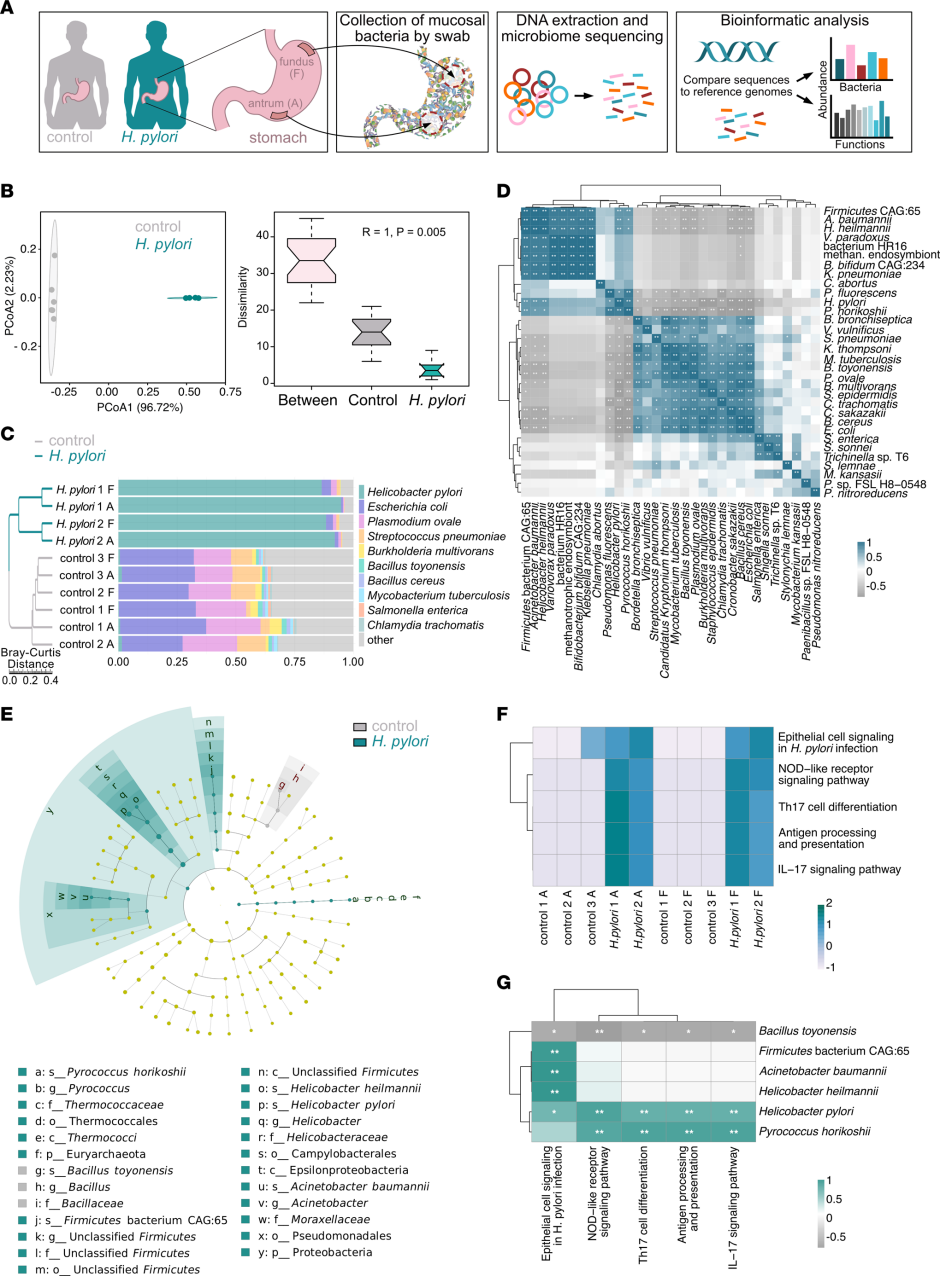
Composition and immune-related functions of the gastric microbiome in HPI tissues. (**A**) Schematic representation of experimental workflow for detecting the gastric microbiome. (**B**) Principal coordinate analysis (PCoA) of identified bacterial species based on Bray-Curtis distance. (**C**) The 10 most abundant bacterial species of the HPI and uninfected tissues. F, fundus; A, antrum. (**D**) Spearman correlation between the identified most abundant species. (**E**) LDA effect size analysis (LDA > 4) of bacteria in the HPI and uninfected tissues. (**F**) Significantly increased microbial functional pathways related to immune function in HPI tissues. (**G**) Spearman correlation analysis between the identified differential species and immune-related KEGG pathways in HPI and uninfected tissues. *P < 0.05, **P < 0.01.

Despite the known gastric microbiome changes related to *H*. *pylori* infection, it is still unclear if the infection affects bacterial species in different gastric regions (5, 6, 13). We collected samples from the fundus and antrum of 5 patients undergoing gastric sleeve surgery, each of whom lacked gastritis-related symptoms. *H*. *pylori* quick-test and culture results showed that 2 patients were HPI at the time of sample collection. Mucosal swab samples then underwent shotgun whole-genome sequencing to functionally characterize the microbiome and describe its bacterial composition (Figure 1A).

The gastric microbiome composition was significantly altered in HPI individuals compared with noninfected individuals (Figure 1B), and the difference between the 2 groups was bigger than the difference within each group (for analysis of similarities: ANOSIM, *P* = 0.005). The metagenomic sequencing data confirmed that infected tissues were heavily dominated by *H*. *pylori*, whereas the uninfected samples had major proportions of *E*. *coli*, *Plasmodium ovale*, and *Streptococcus pneumoniae* (Figure 1C). Concurrent results were observed at the phylum level (Supplemental Figure 1A; supplemental material available online with this article; https://doi.org/10.1172/jci.insight.161042DS1). Correlation analysis illustrated potential mutualistic interactions between *H*. *pylori* and other species of the gastric microbiome. *H*. *heilmannii* and *Acinetobacter baumannii*, which both had increased relative abundances in HPI tissues, were positively correlated with a high relative abundance of *H*. *pylori*, and inversely correlated with the high relative abundance of *Bacillus toyonensis*, which was significantly higher in uninfected tissues (Figure 1, D and E). In addition, *E*. *coli*, *B*. *cereus*, and *Cronobacter sakazakii* had positive relative abundance correlations with *B*. *toyonensis*, but these were all inversely correlated with that of *H*. *pylori*. No heterogeneity in the microbiome was identified between the fundus and antrum samples (Supplemental Figure 1B). These data suggest the potential relationship of different microbes with *H*. *pylori* and the establishment of altered microbiota composition in HPI asymptomatic individuals.

### Gastric microbiome is functionally linked to immune response.

We investigated whether *H*. *pylori*–induced dysbiosis affects the host immune response. Assignment of the shotgun metagenomic reads to the Kyoto Encyclopedia of Genes and Genomes (KEGG) pathway and carbohydrate-active enzymes (CAZymes) databases (14, 15) showed the presence of microbial functions that were evident and distinct between HPI and uninfected tissues (Supplemental Figure 1C).

Microbial function pathways, especially those pertaining to metabolic processes, immune system and infection, were dramatically increased in HPI tissues (Supplemental Figure 1D). Genes conferring glycosyltransferases (GTs) from families GT8, GT9, GT25, and a carbohydrate-binding module (CBM) from CBM50 were significantly increased in HPI tissues (Supplemental Figure 1E), with most enzymes being involved in pathways such as biosynthesis and metabolism of LPS and glycans (Supplemental Figure 1F) (16). Furthermore, all samples from HPI tissues contained the hp1181 gene, which is an important antibiotic-resistance gene associated with the active efflux and multidrug-resistant phenotype (Supplemental Figure 1G) (17, 18). Analysis of immune-related pathways showed a significant increase in epithelial cell signaling, antigen processing and presentation, Th17 cell differentiation and IL-17 signaling, and NOD-like receptor signaling among HPI tissues (Figure 1F). These pathways are widely involved in both B cell and T cell functions. Evaluating correlations between relative abundances for sample microbiota composition and functional pathways also indicated that increased *H*. *pylori* and *Pyrococcus horikoshii* values, along with decreased *B*. *toyonensis* population sizes, were linked to an elevated presence of genes conferring the aforementioned immune pathways (Figure 1G).

*H. pylori* infection leads the influx of immune cells into the gastric mucosa, including plasma cells and Th17 cells (19). The LPS from *H*. *pylori* induces inflammation, and Abs against *H*. *pylori* LPS were observed at high levels in HPI individuals (19, 20). LPS from other bacterial species than *H*. *pylori* also contributes to the interaction with the host immune response (21). Moreover, GT genes from bacteria, including *H*. *pylori*, are essential for glycosylation patterns, which significantly influence the immune recognition and inflammation condition in the gastric mucosa (19, 22). Collectively, these findings suggest the potential for gastric microbiome and immune compartment interplay, with a leading role for *H*. *pylori*.

### scRNA-Seq resolves the lymphoid and myeloid gastric immune compartment.

**Figure 2:**
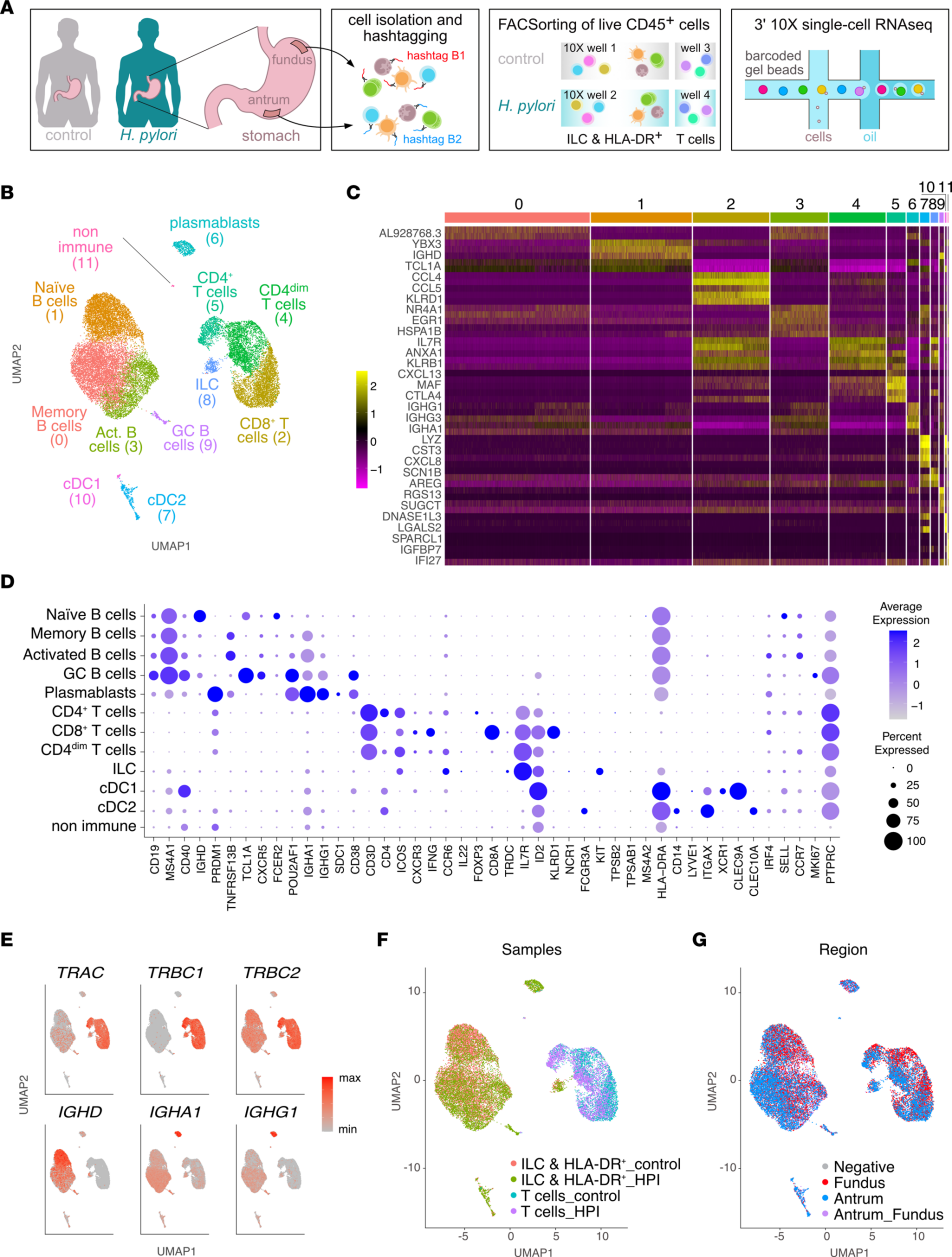
scRNA-Seq resolves the lymphoid and myeloid gastric immune compartment. (**A**) Schematic representation of the experimental workflow leading to scRNA isolation and sequencing of gastric immune cells. (**B**) UMAP showing unbiased clustering analysis of all sequenced cells. (**C**) Heatmap of top differentially expressed genes between clusters shown in **B**. (**D**) Dot plot showing expression of selected cell markers that were used to annotate clusters in **B**. (**E**) UMAP plots showing expression of selected T cell receptor and Ig genes. max, maximum; min, minimum. (**F** and **G**) UMAP visualization of all sequenced cells, color coded on the basis of their origin from each sorted fraction (**F**) and stomach region (**G**). Data represent a pool of cells from the gastric mucosa of 3 HPI and 6 uninfected individuals.

To test the hypothesis that a dysbiotic gastric microbiota associates with an altered gastric mucosal immune compartment, we characterized the immune cell composition of healthy and HPI individuals using scRNA-Seq in an unbiased fashion (Figure 2A). Cells from the fundus and antrum regions of the HPI and uninfected gastric tissues were differentially barcoded with oligonucleotide-conjugated Abs. To enrich low-frequency cell types and allow their transcriptomic profiling, cells were sorted by FACS into 3 main cell populations: Lineage-negative (Lin^–^) CD127^+^ cells (innate lymphoid cells [ILCs]), Lin^+^ cells (T cells), and Lin^–^CD127^–^HLA-DR^+^ cells (containing myeloid and B cells and, henceforth, referred to as HLA-DR^+^) (Figure 2A).

Integration and merging of all data sets resulted in 22,033 high-quality cells (*N* = 19,774 genes expressed in total), which were manually annotated on the basis of signature genes (Figure 2, B–E). Five clusters represented different B cell activation stages (*CD19*^+^*MS4A1*^+^) and plasmablasts (*PRDM1*^+^*CD38*^+^), 3 clusters represented classical T cells (*CD3*^+^*CD4*^+^ or *CD3*^+^*CD8*^+^), 1 cluster represented ILCs (highest average expression of *IL7R* and lack of *CD3*), and 2 clusters were annotated as myeloid cells (*HLA-DRA* and *ITGAX*) (Figure 2D). Annotation of T and B cells was supported by expression of T cell receptor– and Ig-encoding genes, respectively (Figure 2E), and all identified populations belonged to the expected sorted fractions (Figure 2F). Analysis of the fundus- and antrum-associated barcodes revealed no clear separation of cell types based on the stomach region (Figure 2G and Supplemental Figure 2B), which is in line with the nonregionalized microbiome composition previously observed (Supplemental Figure 1B). Thus, scRNA-Seq enabled the visualization of innate and adaptive immune cells in the gastric mucosa.

### Gastric innate cell composition in control and HPI individuals.

**Figure 3:**
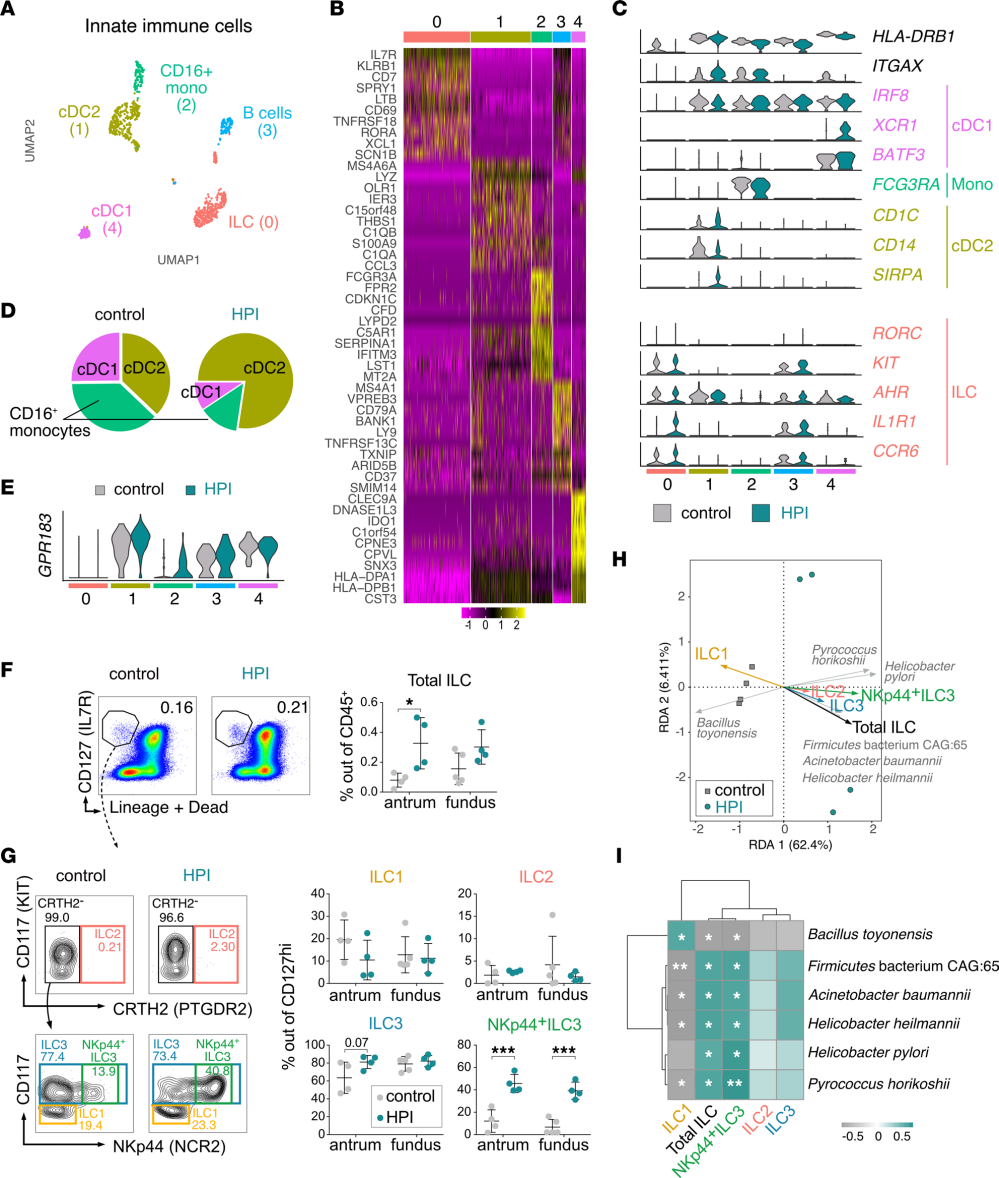
Innate immune cells from the HPI and uninfected tissues. (**A**) UMAP showing unbiased clustering analysis of innate immune cells found in the gastric tissues. (**B**) Heatmap of top differentially expressed genes between clusters shown in panel **A**. (**C**) Violin plots showing expression of selected markers. (**D**) Frequency of each myeloid cell subset within total identified myeloid cells in HPI and uninfected tissues. (**E**) Violin plots showing expression of *GPR183* gene. (**F** and **G**) Representative flow cytometric plots (left) and quantification (right) of total ILC frequency out of CD45^+^ cells (**F**) or ILC subsets (**G**) in the fundus and antrum of HPI (*n* = 4) and uninfected tissues (*n* = 4 antrum, *n* = 5 fundus), based on surface markers indicated in Supplemental Table 3. Data are reported as mean ± SD. *P < 0.05, ***P < 0.001 by 2-way ANOVA with Sidak post hoc test. (**H**) Redundancy analysis (RDA) comparing ILC subset percentages from **G** with the relative abundance of microbial species that were differentially related to control or HPI tissues in Figure 1E. (**I**) Spearman correlation analysis between altered microbial species and ILC percentages induced by *H*. *pylori* infection. *P < 0.05, **P < 0.01.

To further characterize the immune cell compartment in the gastric mucosa, we first focused on the ILC and HLA-DR^+^ samples (Figure 2A and Supplemental Figure 2A). After in silico exclusion of B cells and plasmablasts, the enriched myeloid and ILCs yielded 5 distinct clusters (Figure 3, A and B). Three clusters of myeloid cells were characterized by high levels of *HLA-DRB1* and *ITGAX* expression. Monocytes (cluster 2) were defined by the expression of *FCGR3A* (CD16) and a nonclassical gene expression profile (*CDKN1C*, *LILRB2*) (23), as well as *FPR2*, a marker related to chemotaxis to inflamed tissues (24, 25) (Figure 3C and Supplemental Figure 3A). Conventional type 1 DCs (cluster 4) were identified by *CLEC9A*, *IRF8*, *XCR1*, and *BATF3* expression, whereas conventional type 2 DCs (cDC2s) expressed, among other genes, *CD1C*, *CD14*, and *SIRPA* (Figure 3, A–C, and Supplemental Figure 3A).

*H. pylori* infection affected cell proportions, with cDC2s being the predominant population in the myeloid compartment (Figure 3D). cDC2s from HPI individuals expressed higher *GPR183* (encoding EBI2) levels compared with cDC2s from uninfected gastric tissues, suggesting the formation of ectopic lymphoid follicles (Figure 3E) (26). Cluster 0 was annotated as ILCs, and was mainly was defined by high expression of *IL7R* and *KLRB1* (CD161) (Figure 3, A and B). Among the most differentially expressed genes in the ILC cluster compared with the rest were *SPRY1*, *XCL1*, *LTB*, *CD2*, and *KIT* (Supplemental Figure 3A), which suggests that these cells might contain ILC3 (27, 28).

### ILC3 are the dominant ILC population in the gastric mucosa.

It has been reported that the ILC compartment of the murine stomach is predominantly composed of ILC2s (more than 90% of total ILCs) with virtually no ILC3s (11). To further investigate this, we performed flow cytometric analysis of ILC subsets in the antrum and fundus of 5 uninfected and 4 HPI individuals, according to a well-established gating strategy (28). We found a significantly increased proportion of Lin^–^CD127^+^ ILCs (out of total CD45^+^ cells) in the antrum of HPI as compared with uninfected individuals, with a similar trend, albeit not significant, in the fundus (Figure 3F).

As predicted by the gene expression profile, and in contrast to the mouse data (11), we observed low ILC2 and high ILC3 proportions in the human gastric mucosa, which were unaffected by *H*. *pylori* nor the region analyzed (Figure 3G). *H*. *pylori* infection significantly increased the proportion of gastric NKp44^+^ ILC3s (Figure 3G), which play a role in epithelium defense and tissue remodeling (29). To gain insights into the potential role of the gastric microbiome in affecting ILC homeostasis in the gastric mucosa, we performed redundancy analysis, which shows the correlation between the percentage of ILCs measured by flow cytometry and the relative abundance of each differentially regulated microbial species from the same tissue. *H*. *pylori* and the bacterial species whose abundance increased upon infection (Figure 1E) positively correlated with the proportion of ILC2s, ILC3s, and NKp44^+^ ILC3s (Figure 3, H and I). *B*. *toyonensis* was inversely correlated with ILC2s and ILC3s, but a positive trend with ILC1 percentages was indicated (Figure 3, H and I). Correlation analysis indicated that the gastric microbiome and ILC responses are closely related and potentially influence each other. Altogether, in contrast to mice, the data indicate ILC3s are the dominant ILC population in the human stomach and NKp44^+^ ILC3s are expanded in HPI individuals.

### Enhanced B cell activation in HPI gastric mucosa.

**Figure 4:**
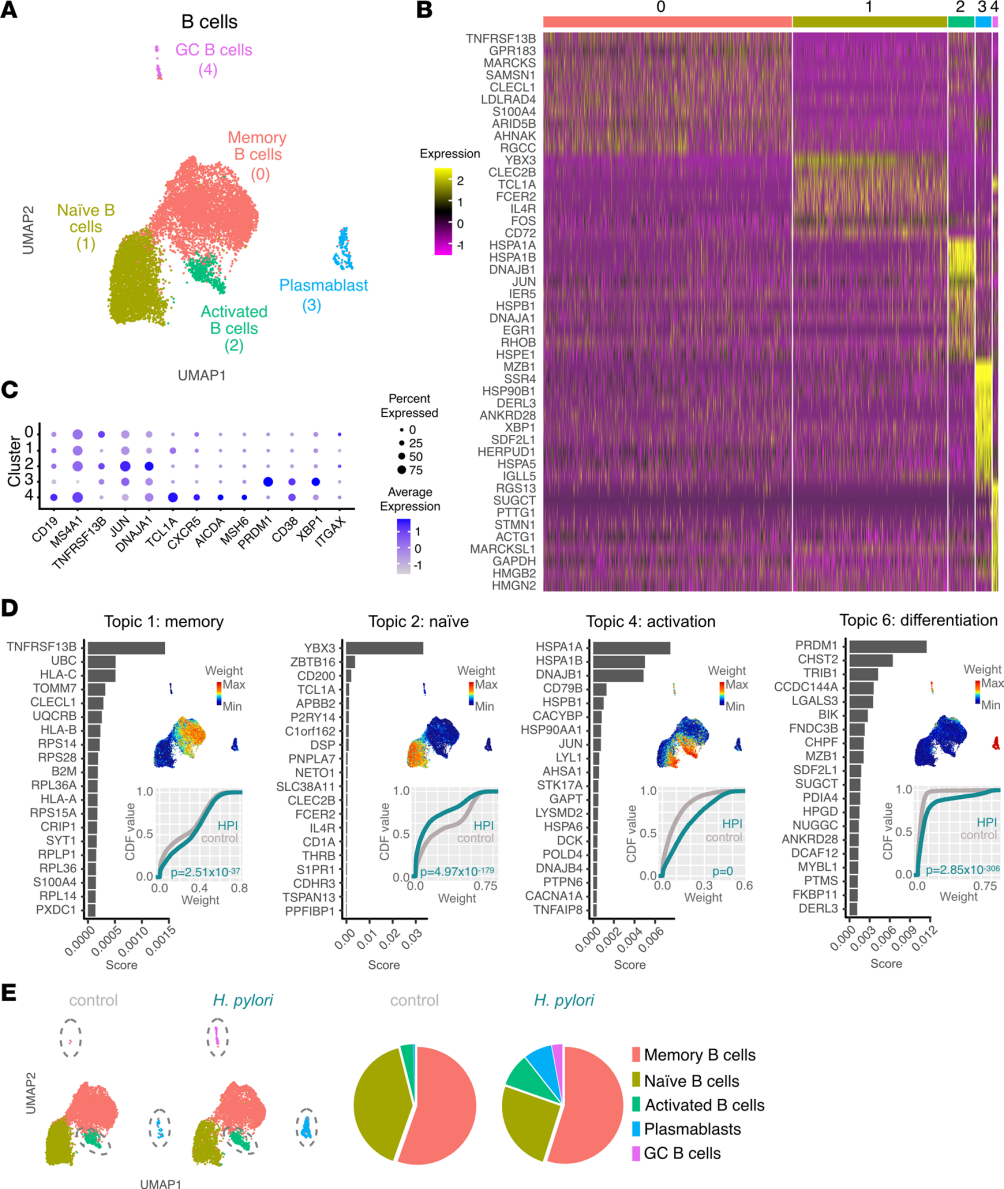
B cell activation and plasmablast differentiation are enhanced in HPI gastric mucosa. (**A**) UMAP showing unbiased clustering analysis of B cell subsets found in the gastric tissues. (**B**) Heatmap of top differentially expressed genes between clusters shown in **A**. (**C**) Dot plot showing expression of selected cell markers. (**D**) Topic modeling of B cell scRNA-Seq data from HPI and uninfected tissues. This includes, for each topic, a bar plot showing the scores (*x* axis) of top-ranked genes within the indicated topic (left); a UMAP plot showing B cells colored by the topic’s weight in the cell (top right); the empirical cumulative density function (CDF) (*y* axis) of topic weights (*x* axis) for the infected and uninfected condition (bottom right). Adjusted *P* values were determined using a Wilcoxon rank-sum test. max, maximum; min, minimum. (**E**) Frequency of each B cell cluster within total B cells in the gastric lamina propria of HPI and uninfected tissues.

We then focused on B cells and plasmablasts (*CD19*^+^*MS4A1*^+^, lacking *CD3D* and *ITGAX* expression) within the ILC and HLA-DR^+^ samples (Figure 2D). Unsupervised clustering yielded 5 cell clusters annotated on the basis of the top differentially expressed genes (Figure 4, A–C). The predominant gastric B cell populations were *TNFRSF13B* (TACI)^+^ *GPR183*^+^ memory (cluster 0) and *YBX3*^+^ *CLEC2B*^+^ *TCL1A*^+^ naive B cells (cluster 1). High expression of *HSP* family genes *JUN* and *EGR1* identified cells in cluster 2 as activated B cells. Cells in cluster 3 were annotated as plasmablasts, on the basis of high expression of *MZB1*, *XBP1*, and the ER chaperones *HSP90B1*, *DERL3*, and *HSPA5* (30).

Finally, we identified cells in cluster 4 as germinal center (GC) B cells, defined by their high expression of *RGS13*, *SUGCT*, *CD38*, and *CXCR5* (Figure 4, B and C) (31). High expression of *AICDA* (or AID) and *MSH6* (32) suggests these cells are undergoing Ig class-switch recombination and somatic hypermutation (Figure 4C).

To further interrogate gene expression programs among gastric B cells in control and HPI mucosa, we applied topic modeling using latent Dirichlet allocations (LDAs) (33, 34). Four of 6 topics described major B cell gene-expression programs within the gastric mucosa (Figure 4D and Supplemental Figure 4, A and B). Topic 1 predominantly weighted within cluster 0 and was defined by the expression of *TNFRSF13B*, *CLECL1*, *CRIP1* (35, 36), and genes encoding for ribosmol proteins, suggesting a relatively quiescent state, thus confirming the identity of memory B cells. Topic 2 heavily weighted within the cells annotated as naive B cells and was defined, among other genes, by *YBX3*, *TCL1A*, and *FCER2* expression. Topic 4 contained genes related to B cell activation, including *CD79B* and *JUN*. Although predominant in activated B cells (cluster 2), topic 4 was also shared by a fraction of naive B cells (cluster 1) and memory B cells (cluster 0) (Figure 3D), indicating the ability of both naive and memory B cells within the gastric mucosa to undergo activation. Topic 6 was enriched in genes related to protein processing and immunoglobulin production (*PRDM1*, *CHST2*, *MZB1*, and *FKBP11*) typical of plasmablasts (37), as well as genes associated with GC reactions (*BIK*, *MYBL1*) (38, 39).

To investigate differences in B cell activity between the HPI and uninfected gastric mucosa, we assessed the weight of these 4 topics in either condition. Topic 2 (naive B cells) had a higher weight in cells from uninfected than HPI tissue. Conversely, topics 1, 4, and 6 (memory, activation, and differentation, respectively) were significantly more prominent in B cells from the HPI gastric mucosa than uninfected gastric mucosa (Figure 4D). Similarly, we detected an increased proportion of activated B cells, GC B cells, and plasmablasts in the HPI sample as compared with uninfected sample (Figure 4E, dashed ovals in the uniform manifold approximation and projection [UMAP]). The expansion of plasma cells in HPI gastric tissues as compared with that of uninfected control samples was confirmed in a previously published single-cell data set encompassing gastric immune cells (40) (Supplemental Figure 4, C–G). Together, these data indicate *H*. *pylori* latent infection in asymptomatic individuals may promote the chronic activation of B cells, formation of GCs, and differentiation of plasmablasts.

### H. pylori infection is associated with reduced CD8^+^ cytotoxic T lymphocytes and increased Th cells.

**Figure 5:**
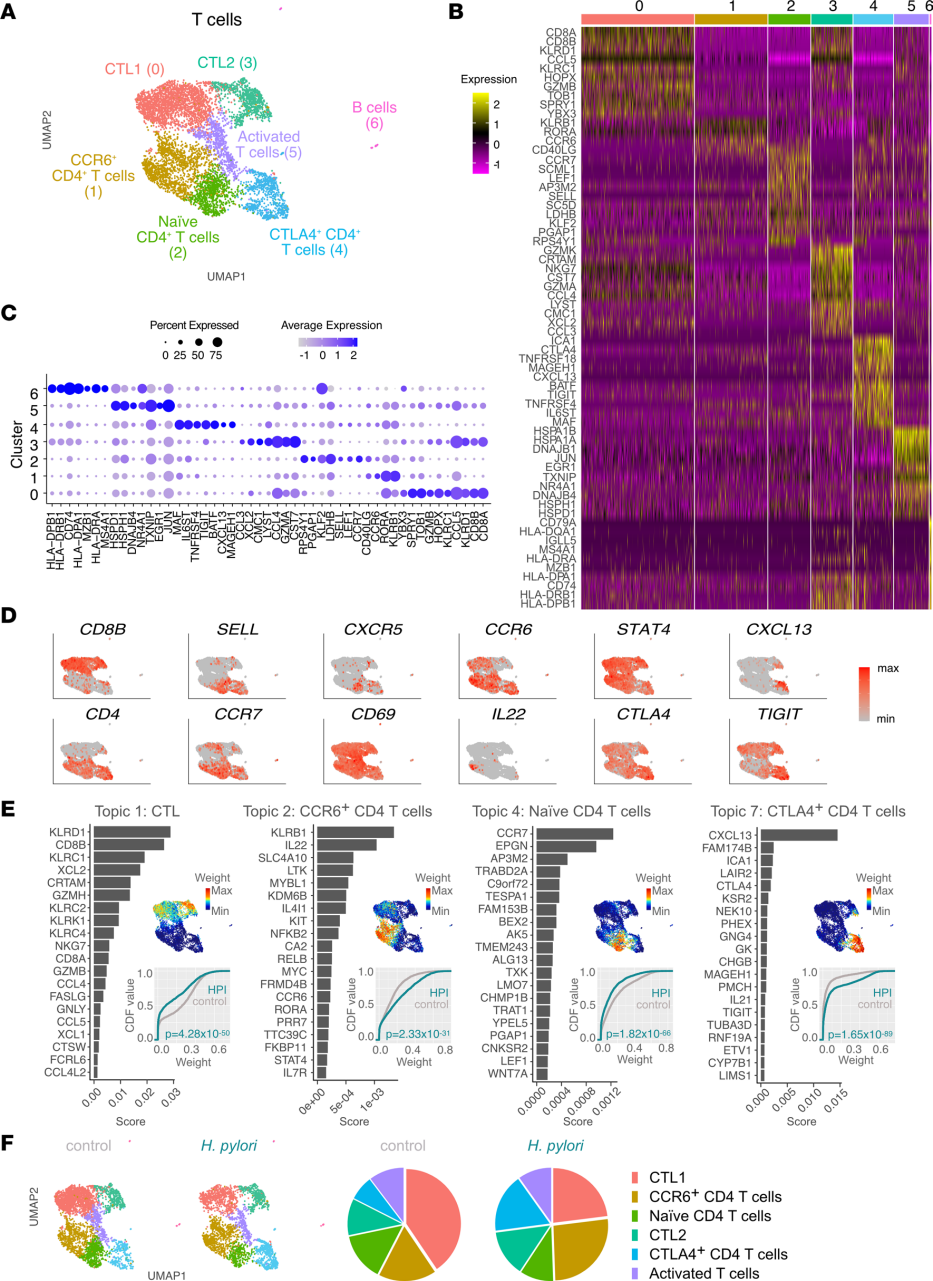
*H*. *pylori* infection is associated with reduced CD8^+^ CTLs and increased Th cells proportions. (**A**) UMAP showing unbiased clustering analysis of T cell subsets found in the gastric lamina propria. (**B**) Heatmap of top differentially expressed genes between clusters shown in **A**. (**C**) Dot plot showing expression of selected cell markers. (**D**) UMAP plots showing expression of selected genes. max, maximum; min, minimum. (**E**) Topic modeling of T cell scRNA-Seq data between HPI and uninfected tissues. This includes, for each topic, a bar plot showing the scores (*x* axis) of top-ranked genes within the indicated topic (left); a UMAP plot showing T cells colored by the topic’s weight in the cell (top right); the empirical cumulative density function (CDF; *y* axis) of topic weights (*x* axis) for the infected and uninfected condition (bottom right). Adjusted *P* values were determined using a Wilcoxon rank-sum test. (**F**) Relative abundance of each T cell subset within total T cells in the stomach of HPI and uninfected tissues.

We then analyzed the T cell compartment, sorted as Lin^+^ (Supplemental Figure 2A). Unsupervised clustering resulted in 6 distinct CD3^+^ T cell clusters (Figure 5, A–D, and Supplemental Figure 5A). Clusters 0 and 3 were annotated as cytotoxic T lymphocytes (CTLs) on the basis of the expression of *CD8A*, *CD8B*, *KLRD1*, and *GZMB*. Additional expression of *GZMK* and *GZMA* suggested that cluster 3 may have an enhanced cytotoxic activity as compared with cluster 0 (41, 42). Cluster 5 grouped early activated CD4^+^ and CD8^+^ T cells characterized by high expression of *HSP*, *JUN*, *EGR1*, and *CD69* (Figure 5, A–D). Clusters 1, 2, and 4 contained CD4^+^ T cells with different degrees of activation. Cluster 1 expressed *KLRB1*, *RORA*, *CCR6*, and *IL22*, which pointed to pro-inflammatory Th cells. Cluster 2 contained *CCR7*^+^*SCML1*^+^*SELL*^+^ naive Th cells. Cluster 4 comprised activated *CTLA4^+^* cells, which expressed a mixed genetic signature pointing into either a Treg (*TNFRSF18*, *MAGEH1*, and *TIGIT*) (43, 44) or follicular helper T (Tfh) direction (*CXCL13*, *BATF*, and *IL6ST*) (45–47) (Figure 5, A–D).

To assess functional differences between T cells in uninfected and HPI conditions, we applied topic modelling to this scRNA-Seq data set, focusing on topics whose weight differed significantly between the 2 conditions (Figure 5E and Supplemental Figure 5B). Topic 1, characterizing a cytotoxic function with genes such as *KLRD1* and *GZMB*, was shared between the previously identified CTL clusters and was more prominent in uninfected than HPI gastric mucosa. Topic 4 (naive CD4^+^ T cells) weight was also reduced in HPI samples. Conversely, topics 2 and 7, encompassing the CCR6^+^CD4^+^ T cluster and CTLA4^+^CD4^+^ T cluster, respectively, were more prominent in the infected condition (Figure 5E). Having defined their gene expression profiles, we also compared proportions of cells within each cluster in control and HPI samples. We observed a decreased proportion of CTL1 and an increased proportion of CCR6^+^CD4^+^ T cells and CTLA4^+^CD4^+^T cells in HPI compared with control individuals (Figure 5F). Altogether, our data suggest an enhanced CD4 Th response in the gastric mucosa of HPI individuals compared with that of noninfected individuals.

### HPI individuals had an enhanced Tfh program in the gastric mucosa.

**Figure 6:**
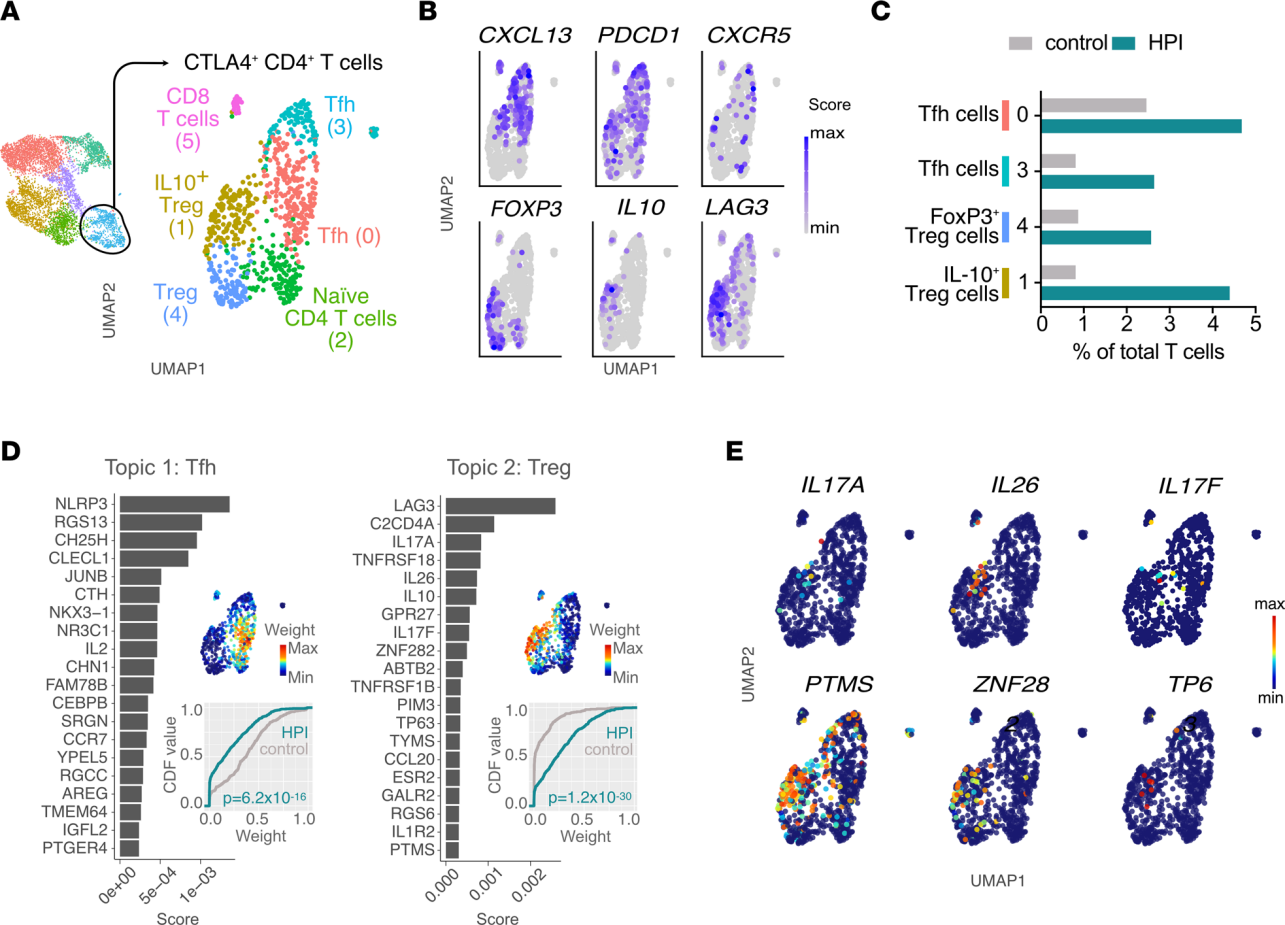
*H*. *pylori* infection is associated with increased Tregs and Tfh cells percentages (implicit, out of total T cells). (**A**) UMAP showing unbiased clustering analysis of CTLA4^+^CD4^+^ T cell subsets found in the gastric lamina propria. (**B**) UMAP plots showing expression of selected genes. (**C**) Frequency of subclusters of CTLA4^+^CD4^+^ T cells out of total T cells in HPI and uninfected tissues. (**D**) Topic modeling of activated CD4^+^ T cell scRNA-Seq data between HPI and uninfected tissues. This includes, for each topic, a bar plot showing the scores (*x* axis) of top-ranked genes within the indicated topic (left); a UMAP plot showing T cells colored by the topic’s weight in the cell (top right); the empirical cumulative density function (CDF; *y* axis) of topic weights (*x* axis) for the infected and uninfected condition (bottom right). Adjusted *P* values were determined using a Wilcoxon rank-sum test. (**E**) Weight of selected genes that define topic 2 in **D**. max, maximum; min, minimum.

To further characterize the CTLA4^+^CD4^+^ T cell population, we subclustered it as shown in Figure 6A, which resulted in 5 CD4^+^ T subclusters. Clusters 1 and 4 were characterized by high expression of Treg-related genes, including *FOXP3*, *IL10*, and *LAG3*, and they were annotated as IL10^+^ Treg and Treg, respectively (Figure 6, A and B). Cluster 2 contained naive T cells, based on the expression of *KLF2* and *RTKN2* (Supplemental Figure 6, A and B) (48). Clusters 0 and 3 were defined as Tfh cells on the basis of the expression of *CXCL13*, *PDCD1*, and *CXCR5* (Figure 6, A and B).

Analysis of subset proportion out of total T cells revealed that proportions of both Tfh and Tregs increased upon *H*. *pylori* infection (Figure 6C). Topic analysis further confirmed the follicular helper and regulatory activities of annotated T cells (Figure 6D and Supplemental Figure 6C). Although the Tfh topic appeared less prominent in HPI than in control CTLA4^+^CD4^+^ cells, the increased proportions of Tfh cells out of total T cells likely indicated a compensatory balance in the infected mucosa. A small portion of cells annotated as IL-10^+^ Tregs, where *FOXP3* was not detected (Figure 6B), coexpressed genes associated with a Th17-type immune response, including *IL17A*, *IL26*, and *IL17F* (Figure 6, D and E) (49), which may hint at the activation of an IL-17 pathway, as inferred by the microbiome data (Figure 1, F and G). Interestingly, the IL10^+^ Treg cluster also expressed high levels of *PTMS*, *ZNF282*, and *TP63* (Figure 6E), which have been associated with oncogene activity and might help explain the association of *H*. *pylori* infection with the development of gastric cancer (50–52). In summary, *H*. *pylori* infection correlates with a reduced CTL response in the gastric mucosa, accompanied by increased CD4^+^ T cell responses, which were characterized by gene expression programs associated with regulatory, oncogenic, and Tfh cell functions.

### Innate and adaptive immune cells interact to form gastric tertiary lymphoid structures during asymptomatic H. pylori infection.

Gastric ILC, cDC2, and B cells had high levels of *LTB* and *GPR183* expression (Figure 3, B and E, and Figure 4B), which pointed to the formation of mucosal tertiary lymphoid structures (TLSs) (53, 54). Human mucosa-associated TLSs are mainly composed of activated or GC B and Tfh cells (55), whose relative abundance was increased in the HPI gastric mucosa. Therefore, we hypothesized the existence of mature TLSs in HPI individuals. To test this, we first inferred paracrine interactions between the identified cell types using SingleCellSignalR analysis of transcriptomic data (56). We quantified the putative ligand-receptor (LR) pairs among B cells, T cells, and innate cell types.

**Figure 7:**
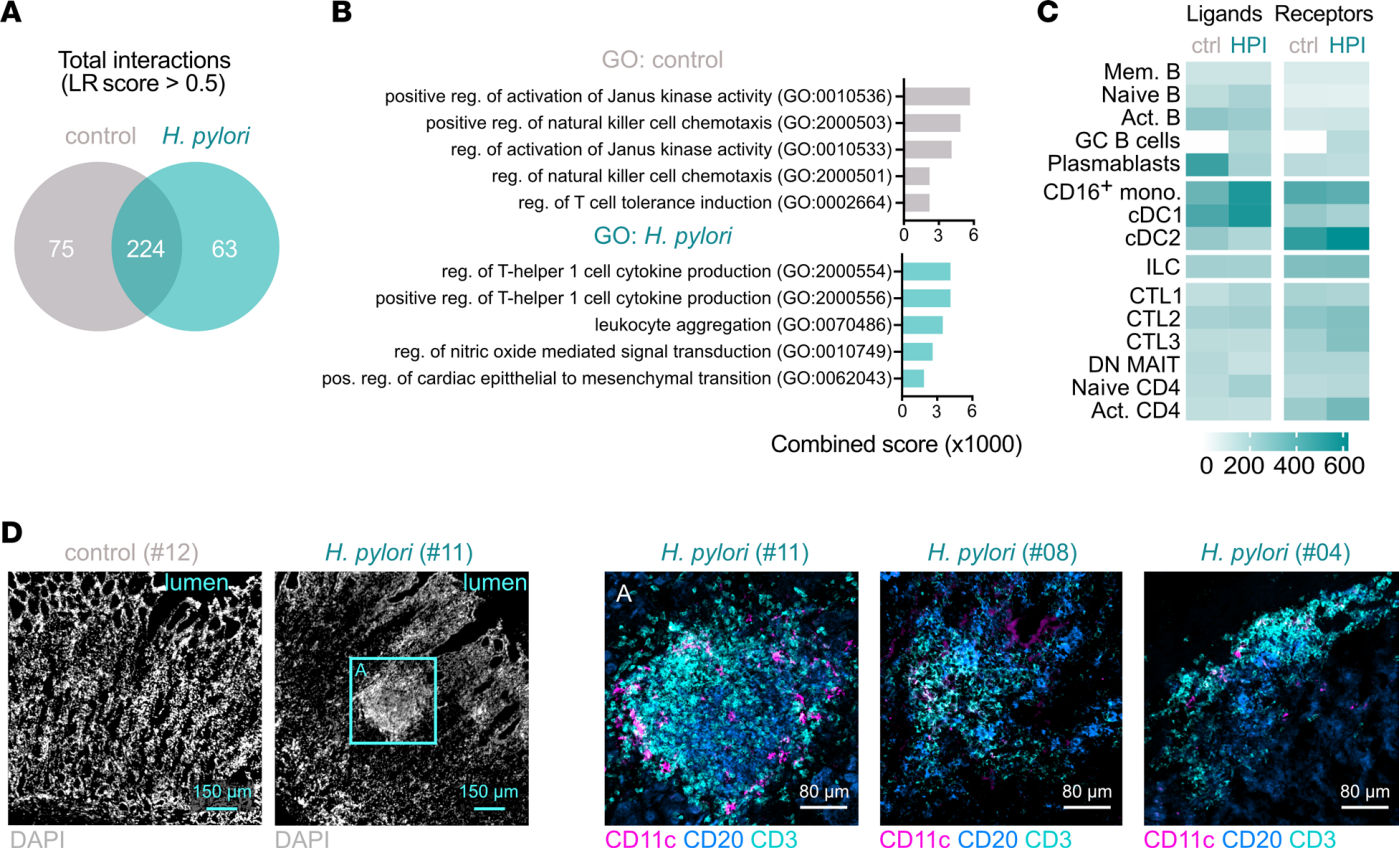
Innate and adaptive immune cells interact to form gastric TLS during asymptomatic *H*. *pylori* infection. (**A**) Number of LR interactions with score > 0.5 predicted by SingleCellSignalR analysis of transcriptomic data of all cell subsets identified in Figure 3A, Figure 4A, and Figure 5A in HPI or uninfected tissues. (**B**) Gene ontology (GO) enrichment analysis of ligands and receptors involved in predicted interactions in **A**. (**C**) Number of putative ligands and receptors offered by any cell type involved in interactions in HPI or uninfected tissues. (**D**) Multicolor immunofluorescence microscopy demonstrating distribution of CD3^+^, CD20^+^, and CD11c^+^ cells within the HPI and uninfected tissues. DN MAIT, double-negative mucosal-associated invariant T; mono, monocyte; pos, positive; reg, regulation; act, activated. Scale bars: 150 μm and 80 μm, as indicated.

Although the overall number of putative interactions with an LR score >0.5 was similar between control and HPI gastric mucosa, 63 interactions (17% of total) only occurred upon *H*. *pylori* infection, and 75 interactions (21%) were specific to the uninfected gastric mucosa (Figure 7A). Gene ontology enrichment analysis of LR pairs indicated that pathways associated with the uninfected mucosa were mostly related to innate immune function and T cell tolerance. Conversely, *H*. *pylori* infection enhanced pathways related to cytokine production and leukocyte aggregation (Figure 7B). Most putative interactions observed in the HPI stomach tissue involved myeloid cells as either ligands or receptors. GC B cells and CTLA4^+^CD4^+^ T cells offered the highest number of receptors in the B cell and T cell fractions, respectively (Figure 7C). In addition, ILCs, cDC2, and CTLA4^+^CD4^+^ T cells from the HPI (but not control) gastric mucosa were predicted to interact via the *LTB*/*LTBR* axis and *VEGFA* angiogenic signals (Supplemental Figure 7, A and B) (57). Therefore, analysis of putative paracrine interactions predicts the possibility that the identified cell types interact with each other to form TLS in the HPI gastric mucosa.

To validate the interaction of B cells, T cells, and CD11c^+^ myeloid cells within TLS in the gastric mucosa, we performed an immunofluorescent costaining of CD20, CD3, and CD11c on uninfected and HPI tissues. All HPI samples analyzed (*n* = 3 distinct individuals), but not control samples, had leukocyte aggregates, usually characterized by a CD20^+^ B cell area surrounded by a CD3^+^ cell region and interspersed CD11c^+^ cells (Figure 7D), indicating the formation of TLS in asymptomatic HPI individuals. Thus, transcriptomic and immunofluorescent data point toward the formation of mature TLS as a hallmark of *H*. *pylori* infection.

## Discussion

By combining microbiome sequencing, scRNA-Seq, flow cytometry, and immunofluorescent microscopy of human gastric tissues, our findings expand current knowledge of the gastric microbiome and immune cell function in uninfected and HPI mucosa. In this study, we showed that asymptomatic *H*. *pylori* infection severely alters the microbiome composition and functional profile of the stomach mucosa. Using scRNA-Seq and flow cytometry, we provided a high-resolution characterization of the human gastric immune cell composition.

Almost all data reported in previous studies of the gastric microbiome were obtained via 16S rRNA gene sequencing; therefore, our use of whole-genome sequencing provides more information at the species level and a glimpse of the corresponding bacterial function. *E*. *coli* and *S*. *pneumoniae*, the predominant species composing the gastric microbiome in control samples, often were found in other studies (5, 6). Also in line with findings of previous studies, we demonstrated a significant shift of the gastric microbiome composition after *H*. *pylori* infection (6, 58–61). We did not find significant changes of the gastric microbiome between antrum and fundus, expanding previous knowledge obtained from a comparison of the antrum and corpus regions (62). Notably, *P*. *ovale* was detected in the samples, which we believe probably is due to the sequences of the short reads mapped to the environment bacteria. Preliminary analysis of the metagenomes using Kraken2 and the Genome Taxonomy Database led to the classification of many bacterial taxa that are commonly observed in diverse environmental samples and, consequently, resulted in a much broader microbial community composition than that described for the human gut microbiome. In response, we elected to characterize the stomach microbiome samples using the well-established method in conjunction with the National Center for Biotechnology Information NR database described in Methods. In addition, we detected a low abundance of *H*. *pylori* in the control samples. The bacteria were either dead or the activity was under the detection level of the *H*. *pylori* quick-test kit. A similar observation was reported previously with metatranscriptomic RNA-Seq (63).

Moreover, we observed a significant increase of the genes encoding for enzymes involved in LPS and glycan biosynthesis and metabolism within the HPI tissues. LPS is actively involved in the immune response via modulation of antigen processing and presentation (64), the Th17 pathway (65), and the NOD-like receptor signaling pathway (66). Glycans also serve as key checkpoints of T cell function (67). These findings are further supported by our metagenomic pathway analysis and corresponding immune survey. The alteration of the gastric microbiome genes and immune signaling pathways detected in our study are in line with findings from other groups investigating gastritis and gastric cancer (59–61). Although the high predominance of *H*. *pylori* may suggest it is the lead player in eliciting immune activation and subsequent gastritis, the changes in the immune response may also result from the alteration of the whole gastric microbiome. The correlation between gastric microbes other than *H*. *pylori* and immune pathways provided evidence for this hypothesis and further prompted us to investigate the effect of such a dysbiotic microenvironment on the immune compartment in the underlying gastric mucosa.

Our scRNA-Seq analysis of the B cell compartment revealed that *H*. *pylori* infection promoted B cell (re)activation, GC formation, and differentiation of plasmablasts, which were hardly detectable in uninfected tissues. These findings are in line with previous reports of B cell follicle formation and plasma cell accumulation in the gastric lamina propria of HPI patients and mice infected with *Helicobacter* spp. (68, 69).

We described a shift in the balance between CD8^+^ CTL and CD4^+^ T cells in HPI tissues as compared with uninfected tissues. An enhanced proportion of Tfh cells, although potentially less functional, is in line with the increased proportion of GC B cells detected in the infected tissues. Notably, activation of the Tfh program is not impaired but rather coexists with the immunosuppressive function of Tregs that have been linked to evasion of *H*. *pylori* from immune-mediated clearance mechanisms and protection from peptic ulcer (70–72). Our observations thus support the hypothesis of an equilibrium between an immunosuppressive environment and B cell expansion in the establishment of asymptomatic *H*. *pylori* infection in the human gastric mucosa, as was previously suggested in mice (73). Although decreased CTL function fits an immunosuppressive environment, understanding the potential role of increased CCR6^+^CD4^+^ T cells upon *H*. *pylori* infection requires additional insight.

Numerous studies have investigated the immune response against persistent microbial stimulation of the intestinal mucosa and the formation of TLS in the intestine, but rarely has this been studied in the human stomach. Interestingly, the transcriptional profile of cDC2s that were predominant among myeloid cells in the HPI gastric mucosa resembled that of DCs found in association with intestinal cryptopatches (74). Similarly, ILC3s promote mucosal Ab responses in the murine intestine (75, 76), and we also found that NKp44^+^ ILC3s were increased in HPI as compared with uninfected individuals. These cells play an important role in epithelium defense and tissue remodeling in the small intestine (29), and more studies are needed to assess whether they could exert a similar function in the gastric mucosa.

To our knowledge, this is the first characterization of the ILC compartment in the human gastric mucosa by both scRNA-Seq and flow cytometry. In an elegant study, investigators recently reported that the murine stomach contains predominantly ILC2s, which promote *H*. *pylori*-specific IgA responses upon infection (11). The apparent discrepancy between this murine study and our observations in human tissue could be explained by several factors, including acute versus chronic type of the infection, host variation, microbiome, and diet differences, and other environmental exposures. Moreover, ILC2s recently have been shown to undergo transition to an ILC3-like state under certain inflammatory conditions, which would support a less compartmentalized description of the ILC of the stomach (34, 77).

One limitation of the present study is small sample size. Despite this, we performed multiple investigative approaches on the same human gastric tissues, which provided novel correlations and predicted interactions between gastric microbiome and immune cells. Second, the tissues were obtained from obese individuals, mainly female, which might influence the generalization of our findings to healthy male individuals. However, this setting gave us the unique chance to analyze gastric tissue from HPI individuals without clinical manifestations of gastritis. Also, our data were generated by comparing tissues with and without *H*. *pylori*, both on the obese background, which should minimize the confounder factor derived from obesity and make our findings on *H*. *pylori*–related changes in the microbiome and immune response solid. In addition, this is not a complete immune cells survey; we left out some immune cells, such as granulocytes and NK cells, from scRNA-Seq analysis. Additional studies are needed to prove if *H*. *pylori* infection is the cause of all the changes observed in the gastric microbiome and immune cells.

### Conclusion.

By combining microbiome sequencing, scRNA-Seq, flow cytometry, and immunofluorescent microscopy of human gastric tissues, our findings expand current knowledge of the gastric microbiome and immune cell function in uninfected and HPI mucosa. A deeper analysis of the interactions between the microbiome and innate and adaptive immune cells in the context of active gastric pathology, as compared with asymptomatic infection, that we hereby performed may be instrumental to develop new therapeutic approaches for the treatment of less benign infections of the stomach and their severe outcomes.

## Methods

### Study population.

In total, 24 samples were obtained from 14 individuals undergoing sleeve gastrectomy for morbid obesity at Ersta Hospital in Stockholm, Sweden. Samples were processed within 40 minutes. The BIOHIT *H*. *pylori* quick-test kit (Biohit HealthCare) was used to detect infection in 2 representative pieces of fundus and antrum. HPI status was confirmed by culturing swab samples from 28 to 32 gastric sites on GC agar plates (Karolinska Hospital, catalog MIK0346) under microaerophilic condition. Characteristics of the participants, based on questionnaire data, are listed in Supplemental Table 1.

Epithelial microbiome samples were collected from fundus and antrum using swabs (FLOQSwabs, Copan Flock Technologies), and stored in DNA/RNA shield (Zymo Research) at –80°C until DNA extraction and metagenome sequencing. Fundus and antrum tissues were processed for isolation of mononuclear cells. Antrum and fundus tissues (approximately 1 cm^2^) were snap-frozen for immunofluorescence microscopy.

### Metagenome sequencing.

Stomach microbiome samples (*n* = 10: 4 HPI and 6 uninfected) were bead-beaten with Matrix E beads (MP Biomedicals) for three 2-minute intervals and then incubated with lysozyme (100 mg/mL; Sigma-Aldrich) at 37°C for 60 minutes followed by digestion with 20 μL proteinase K (20 mg/mL; Qiagen) at 55°C and 250 rpm for 90 minutes. Data extraction steps were performed with DNeasy Blood & Tissue Kit (Qiagen) according to the manufacturer’s guidelines. Metagenomic sequencing and analysis were performed by Novogene using a standard protocol for low-input samples. Sequencing was performed on Illumina NovaSeq/Hiseq Xten (Illumina) using NovaSeq Reagent Kits/HiSeq X Reagent Kits according to the manufacturer’s instructions.

### Analysis of metagenome sequencing data.

Low-quality reads (*Q* ≤ 38, which exceeds 40 bp), number of records, reads overlapping with adapter, and human reads together with their mated or paired reads were removed using Soap 2.21 (78) and assembled using an optimized SOAPdenovo protocol (79). MetaGene was used to predict ORFs from the assembled contigs with length longer than 100 bp. Assembled contigs were then pooled and nonredundancies were constructed using cluster database at high identity with tolerance (CD-HIT) with 95% identity (80). We mapped original sequences to the predicted genes and estimated the abundances using SOAPaligner (81). Taxonomic assessment was performed using MEGAN (82) against the sequences of bacteria, fungi, archaea, and viruses extracted from the National Center for Biotechnology Information nonredundant protein (NCBI NR database (version 2016-11-05). Taxonomic profiles were conducted with relative calculated abundance. Very low abundance of *Candidatus* Kryptonium thompsoni, which was found at the same level in all the samples, was considered contamination and removed from the analysis. Abundances of KEGG orthology pathway and KEGG enzyme modules, CAZymes, and antibiotic-resistance genes were annotated on the basis of KEGG, CAZymes, and Comprehensive Antibiotic Research databases (15, 83, 84), and normalized into counts per million reads for downstream analysis.

### Isolation of mononuclear cells from the gastric lamina propria.

Gastric tissue (approximately 1–5 g) was cleaned from fat, cut into 0.5 cm pieces, and incubated for 30 minutes at 37°C in HBSS with 5% FCS, 5 mM EDTA, 1 mM DTT, and 15 mM HEPES under gentle shaking. The tissues were washed with PBS plus 5% FCS and 1 mM EDTA at 37°C followed by washing in PBS plus 1% FCS and 15 mM HEPES at room temperature. Cell suspension was obtained by digestion in serum-free HBSS with Collagenase D (0.5 mg/mL; Roche) and 0.1 mg/mL DNase I (Roche) at 37 °C and 600 rpm for 35 minutes, then filtered (using a 100 μm cell strainer) and separated with a 44%–67% Percoll (Sigma-Aldrich) gradient. Cells were immediately used or frozen in FCS plus 10% DMSO.

### Cell sorting prior to scRNA library preparation and flow cytometry.

Frozen cells were thawed in a 37°C water bath. Samples from the same group (i.e., stomach region, HPI status) were pooled and washed in warm complete RPMI plus 10% FCS. Cells were then stained with hashtag Abs (B1 for fundus and B2 for antrum) and staining Abs (Supplemental Table 2) for 30 minutes. Cells were extensively washed and sorted on a SH800S sorter (Sony) with a Purity sorting mask. Cells were sorted as single DAPI^–^CD45^+^Lin^+^ (T cells), DAPI^–^CD45^+^Lin^–^CD127^+^ (ILCs) and DAPI^–^CD45^+^Lin^–^CD127^–^HLA-DR^+^ (B cells and myeloid cells). For flow cytometry analysis, samples were stained with the Abs listed in Supplemental Table 3. An LSR Fortessa flow cytometer (BD Biosciences) was used for acquisition, and data were analyzed with FlowJo software (Tree Star).

### scRNA isolation and sequencing.

Samples were processed with Chromium Single Cell 3′ Reagent Kits, version 3, with feature barcoding technology for cell surface protein (10x Genomics) according to the manufacturer’s instructions. Samples were sequenced at Novogene.

### Analysis of scRNA-Seq data.

Data were preprocessed with the 10x Genomics Cell Ranger 3.1.0 pipeline. The Seurat 3.1.3 R package (85) was used for downstream single-cell clustering and differential expression analysis. First, we aligned the reads from stomach single-cell data sets on the reference hg19 (for all analyses in this article) or Grch38 (hg38) (to specifically interrogate expression of TCR and Ig genes shown in Figure 2) genomes using the Cell Ranger pipeline for the quantification of the cells as well as genes and cell surface proteins. We performed quality check and a filtering protocol based on cutoff criteria for cells (number of feature expressions per cell >200 and <2500); gene expression (≥3 cells), mitochondria content (<10 %), ribosomal content (>5%), regression of cell cycle effect, and removing the noncoding gene from the expression data set. A total of 22,033 cells with 19,774 genes expressed in total were analyzed and visualized using UMAP and t-distributed stochastic neighbor embeddings. Topic modeling was performed as described elsewhere (33). Interactome analysis was performed using the SingleCellSignalR package (56).

### Multicolor immunofluorescence microscopy.

Biopsy specimens were embedded in Tissue Tek OCT medium and snap frozen in dry ice before storage at –80°C. Sections (8 μm) were mounted on Superfrost plus glass slides (Thermo Fisher Scientific) and kept at –80°C. Staining was performed as described elsewhere (86), with minor modifications, using the Abs listed in Supplemental Table 4. All slides were mounted with Fluoromount Aqueous Mounting Medium (Sigma-Aldrich) and images were acquired on an LSM 700 system (Carl Zeiss) equipped with 405, 488, 555, and 639 nm excitation lines at the Biomedicum Imaging Core at the Karolinska Institute. Images were analyzed using Zen 2.3 software (Carl Zeiss) or the Imaris Viewer 9.5.1 (Bitplane).

### Statistics.

Spearman correlation was used when the most abundant species were identified and when the identified differential species and immune related KEGG pathways in HPI and uninfected tissues were compared. Analysis of similarities was applied to compare the similarities between the fundus and antrum regions, as well as between the functional abundance of the HPI and uninfected tissues. Metastats analysis with a *q* value <0.05 was used to analyze metagenome functions, metagenome enzymes, and genes. Significantly changed microbial CAZymes families was found using LDA effect size analysis (LDA > 3). Flow cytometry data are presented as mean ± SD. Statistical analysis was determined using GraphPad Prism, version 7.0. Statistical significance was determined by ordinary 2-way ANOVA with Sidak post hoc test. *P* < 0.05 was considered statistically significant. Spearman correlation analysis was performed between bacterial species and immune-related KEGG pathways or ILC proportions.

### Data transparency.

Nucleotide sequencing data are made available in public, open-access repositories. Shotgun metagenomic DNA sequences were deposited in the European Nucleotide Archive under project accession number PRJEB48730. scRNA-Seq data were submitted to the European Nucleotide Archive under project accession number PRJEB48798.

### Study approval.

The study was approved by the Regional Ethical Board at Karolinska Institute, Stockholm, Sweden (no. 2016/573-31). Participants gave their consent before sample collection.

## Author contributions

CS, LE, JD, and EJV conceived of the study. CS, KPT, SW, SMH, JW, LC, SB, AR, TK, JD, and EJV curated the data. AT, LE, JD, and EJV contributed to investigation and funding acquisition. CS, KPT, SW, and SMH contributed to the study methodology and formal analysis. Project administration was conducted by JD and EJV. AT, LE, JD, and EJV contributed to study resources. AT, LE, JD, and EJV supervised the study. Visualization was performed by CS, KPT, and SW. CS, KPT, SW, JD, and EJV wrote the original draft of the manuscript; all authors reviewed and edited the manuscript and had access to the study data and approved the final version of the manuscript.

## Supplementary Material

Supplemental data

Supplemental table 5

## Version 1 02/22/2023

Electronic publication
