# Supplementary material for: Metagenomic and single-cell RNA-Seq survey of the Helicobacter pylori–infected stomach in asymptomatic individuals
Source: JCI Insight. 2023 Feb 22;8(4):e161042. doi: 10.1172/jci.insight.161042 (PMC9977493; doi:10.1172/jci.insight.161042)
Supplement: Supplemental data [file jciinsight-8-161042-s032.pdf]

SUPPLEMENTAL MATERIAL

Metagenomic and single-cell RNA-seq survey of the *H. pylori*-infected stomach in asymptomatic individuals

Short Title: Microbiome and immunology survey of stomach

Chiara Sorini[1,2,*], Kumar P. Tripathi[1,2,*], Shengru Wu[3,*], Shawn M. Higdon[3], Jing Wang[3], Liqin Cheng[3], Sanghita Banerjee[1,2], Annika Reinhardt[1], Taras Kreslavsky[1], Anders Thorell[4], Lars Engstrand[3], Juan Du[3,§,#] and Eduardo J. Villablanca[1,2,§,&,#]

[1] Division of Immunology and Allergy, Department of Medicine Solna, Karolinska Institutet and University Hospital, Stockholm, Sweden

[2] Center of Molecular Medicine, Stockholm, Sweden

[3] Department of Microbiology, Tumor and Cell Biology, Centre for Translational Microbiome Research (CTMR), Karolinska Institutet, Stockholm, Sweden

[4] Ersta Hospital, Stockholm, Sweden

* and § These authors contributed equally to this work

#Correspondence: J.D. (juan.du@ki.se) and E.J.V. (eduardo.villablanca@ki.se)

[&] Lead contact E.J.V. (eduardo.villablanca@ki.se)

SUPPLEMENTAL FIGURES

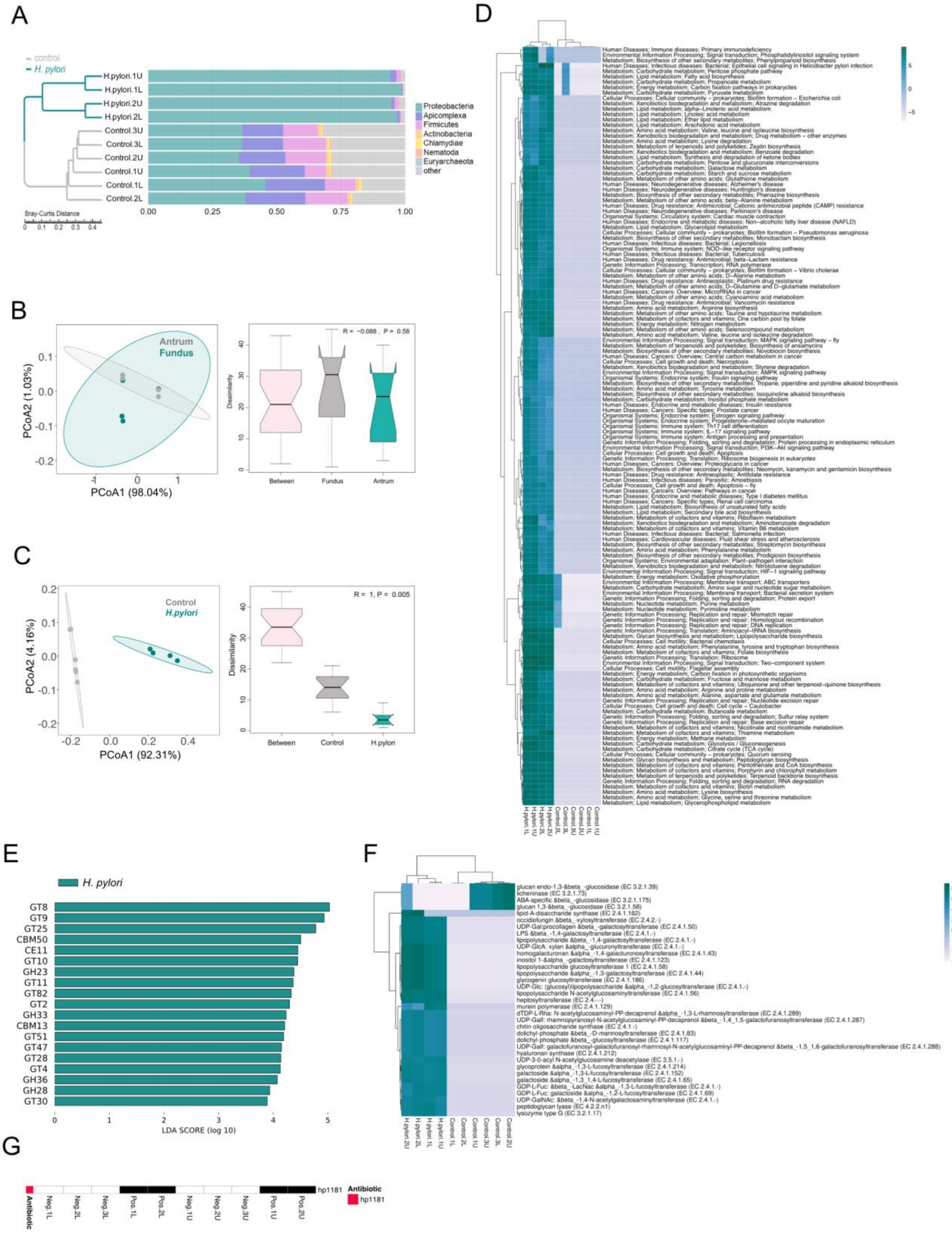

Legend on the next page

Suppl. Figure 1 | Composition and function of the gastric microbiome are altered in H. pylori-infected tissues.

a. The ten most abundant bacteria at the Phylum level and sample clustering of the HPI and uninfected tissues based on Bray-Curtis distance. **b.** The PCoA plot of fundus and antrum samples with identified bacterial species based on Bray-Curtis distance. The analysis of similarities (Anosim) showing no difference in the microbial diversity between the fundus and antrum regions. **c.** The PCoA plot based on functional abundance in KEGG database. The analysis of similarities (Anosim) showed a significant difference in the functional abundance of the HPI and uninfected tissues. **d.** Heatmap highlighting the significantly altered microbial functional pathways in KEGG database between HPI and uninfected tissues. The differential metagenome functions were obtained using Metastats analysis with *q* value < 0.05. **e.** Significantly changed microbial carbohydrate-active enzymes families from the gastric microbiome of the HPI tissues using linear discriminant analysis (LDA) effect size (LEfSe) analysis (LDA>3). **f.** Significantly changed enzymes observed from the gastric microbiome of the HPI tissues compared to uninfected tissues from KEGG enzyme database. The differential metagenome enzymes were obtained using Metastats analysis with *q* value < 0.05. **g.** Only resistance gene hp1181, expressed in the *H. pylori*-infected tissues, was identified based on the comprehensive antibiotic research database (CARD). The differential metagenome gene was obtained using Metastats analysis with *q* value < 0.05.

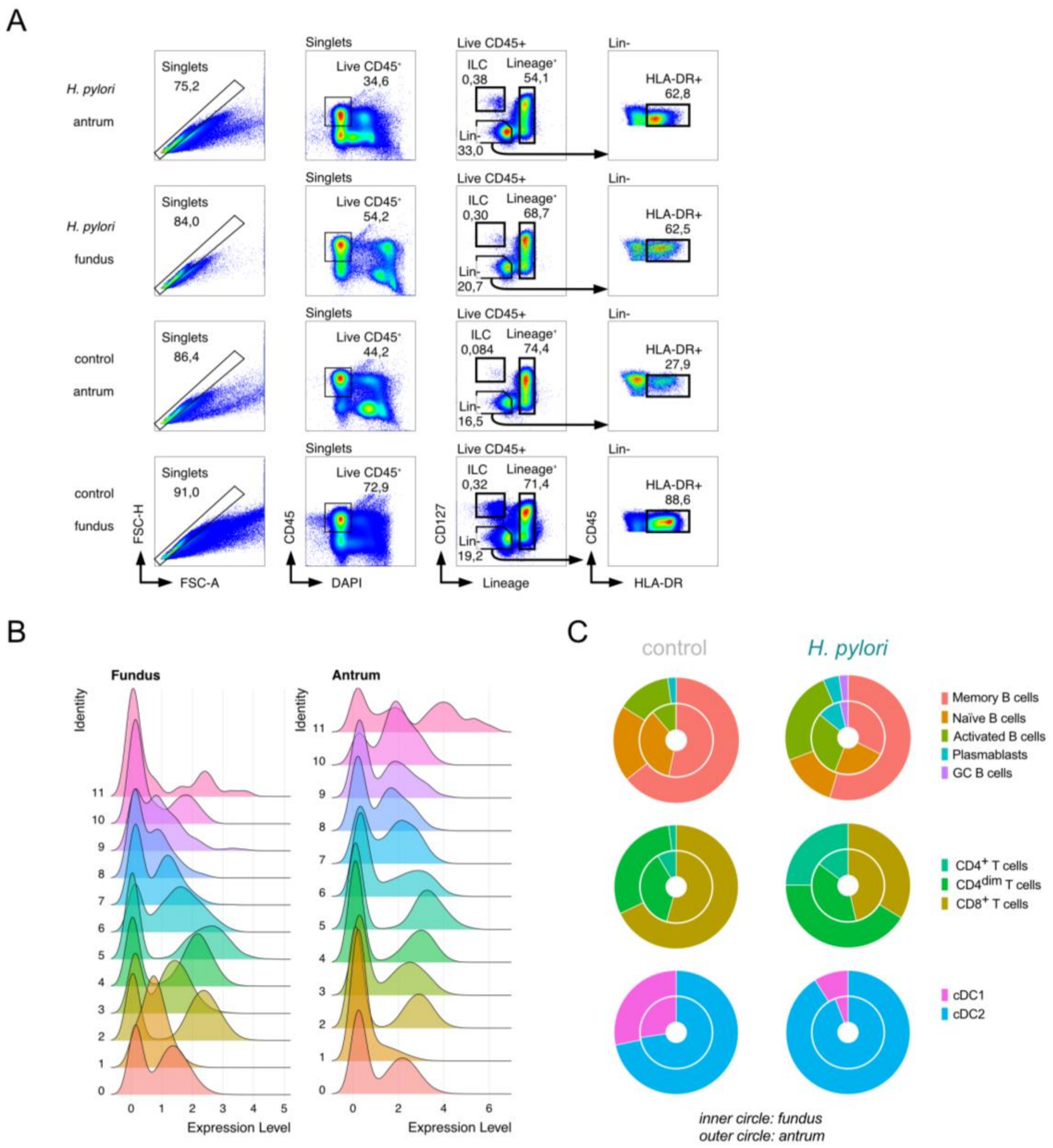

Suppl. Figure 2 | Sorting and clustering of immune cells from the stomach of HPI and uninfected tissues.

a. Gating strategy for the sorting of selected immune cells from the stomach of HPI and uninfected tissues. **b.** Histograms showing expression of fundus- and antrum-associated hashtags within each cluster identified in Figure 2b. **c**. Pie charts comparing percentages of clustered immune cells from Fig. 2B in antrum and fundus, within the three major groups of co-enriched cells: B cells, T cells and myeloid cells.

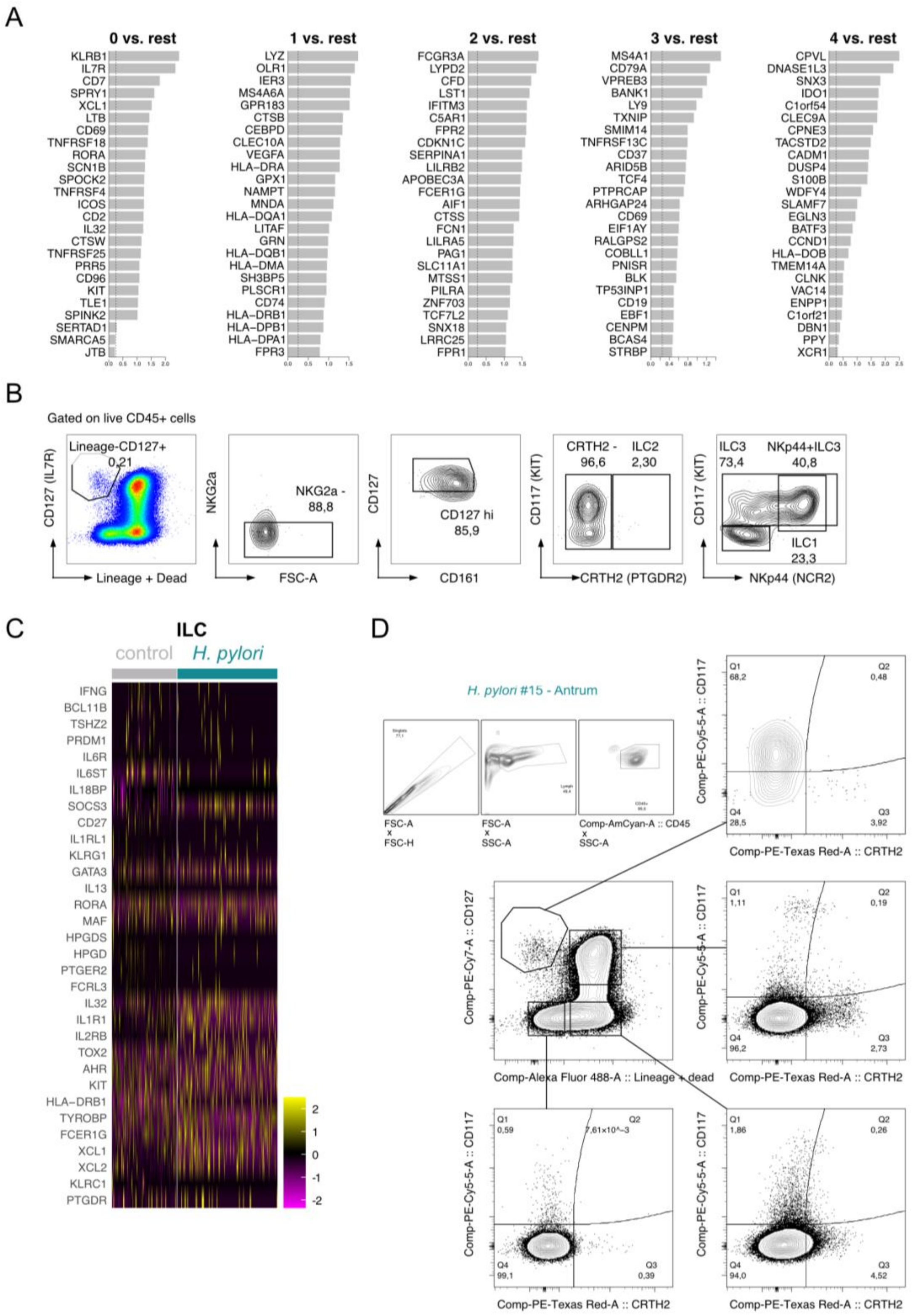

Suppl. Figure 3 | Innate immune cells from the HPI and uninfected tissues.

a. Top differentially expressed genes in clusters shown in Figure 3a. **b.** Complete gating strategy used for the identification of ILC subsets in the gastric lamina propria. **c.** Transcriptomic insight of cells annotated as ILC in Figure 5a. **d.** Example of CD117 and CRTH2 protein expression in $CD45^{+}$ cells other than $Lineage^{-}CD127^{+}$ ILCs.

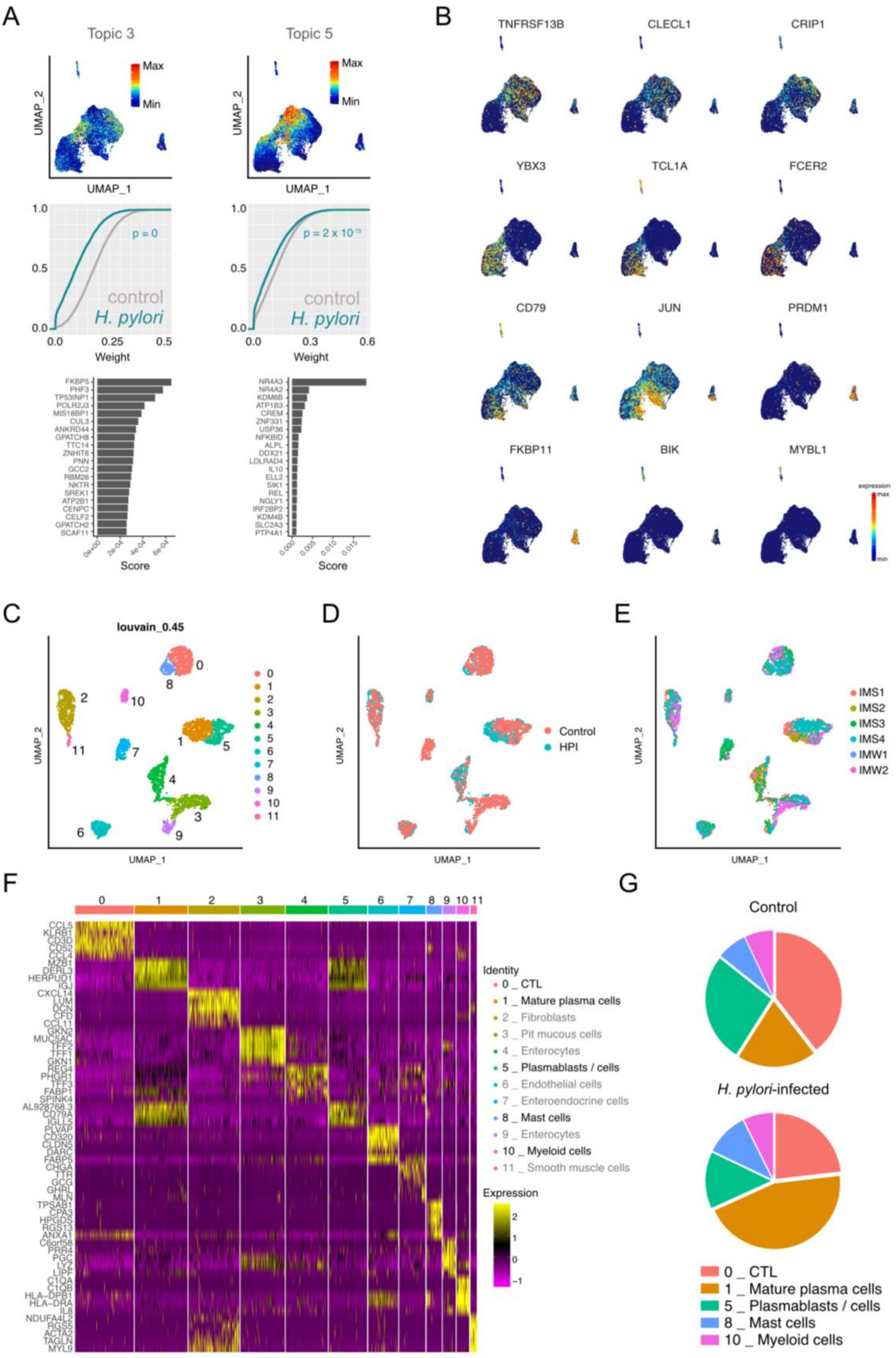

Legend on the next page

Suppl. Figure 4 | Additional B cell topic analysis in the HPI and uninfected tissues. **a**. B cell topics left out from analysis in Figure 4d. **b**. Weight of selected genes that define B cell topics. **c-e**. UMAP visualization of gastric cells from intestinal metaplasia patients sequenced by Zhang *et al.*, *Cell Rep* (2019) (GSE134520) (40), color-coded based on unbiased clustering (c), *H. pylori* status (d), and their sample origin in the published paper (e). **f**. Heatmap of the clustered GSE134520 dataset. **g**. Percentages of immune cells detected in the clustered GSE134520 dataset.

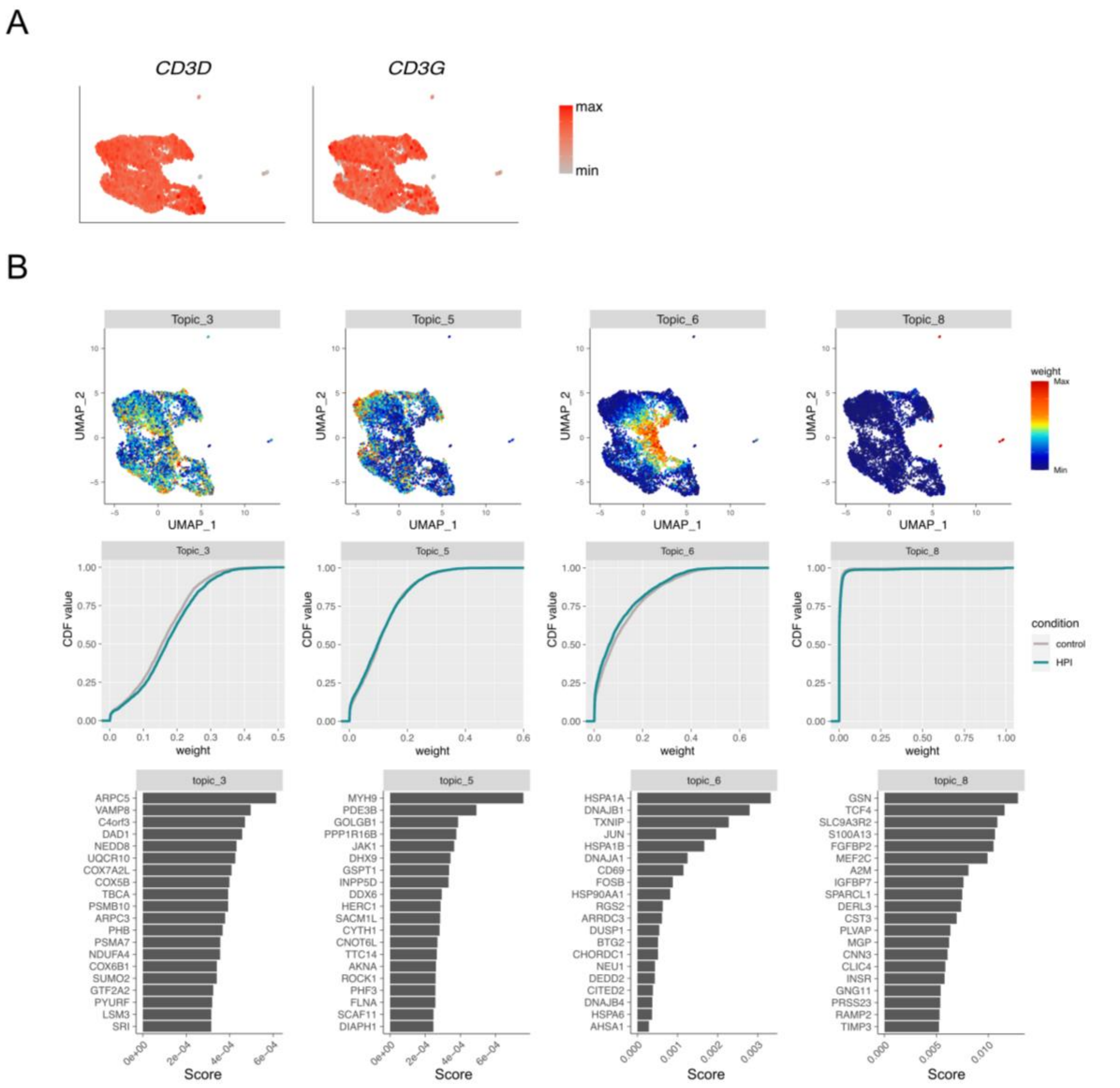

Suppl. Figure 5 | Additional T cell subset and topic analysis in the HPI and uninfected tissues.

a. UMAP plots showing expression of *CD3D* and *CD3G* in T cells. **b.** T cell topics left out from analysis in Figure 5d.

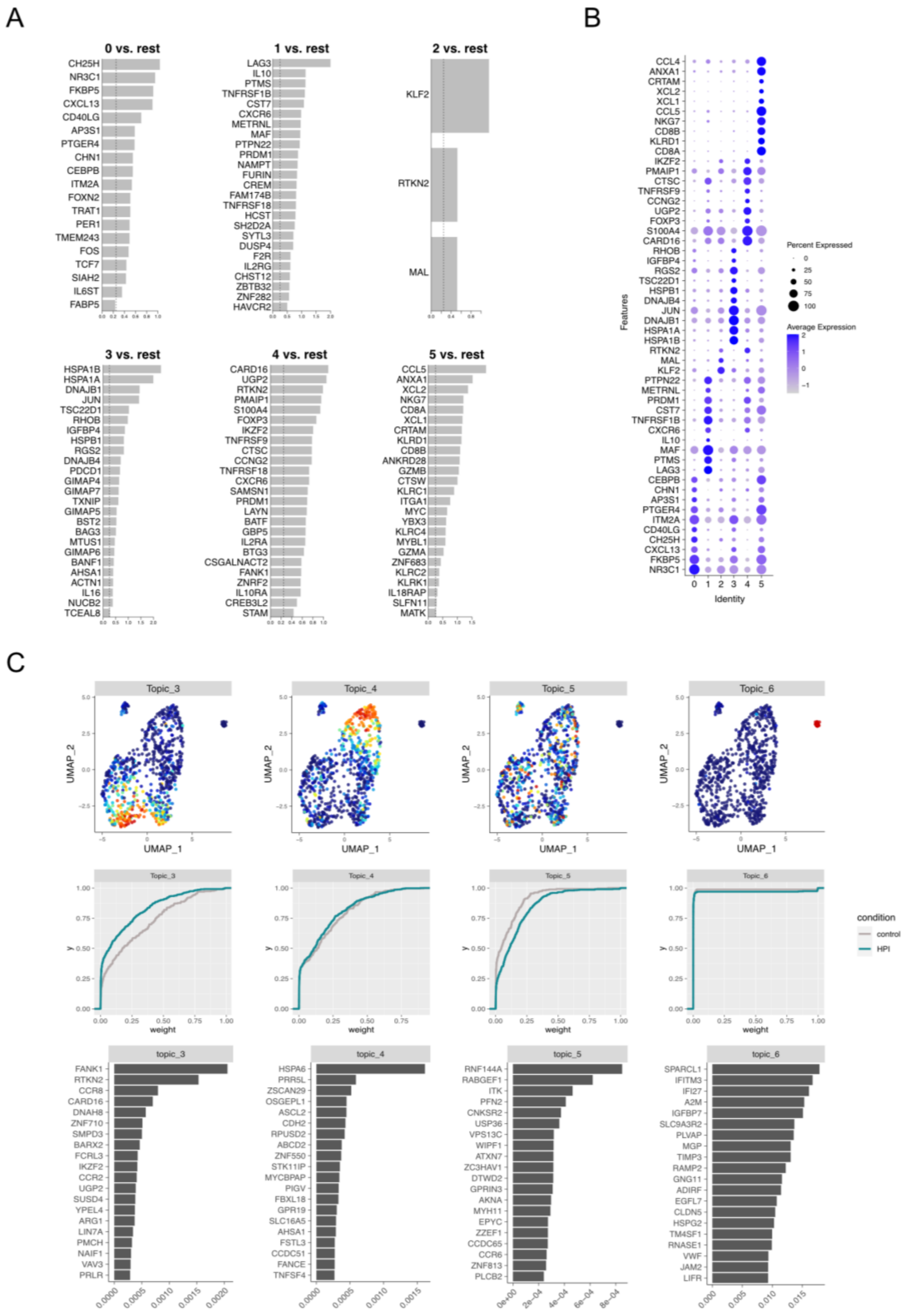

Suppl. Figure 6 | Characterization of activated CD4+ T cell subsets in the HPI and uninfected tissues.

a. Top differentially expressed genes in clusters shown in Figure 6a. **b.** Dotplot showing expression of selected cell markers that were used to annotate clusters in Figure 6a. **c.** T cell topics left out from Figure 6d.

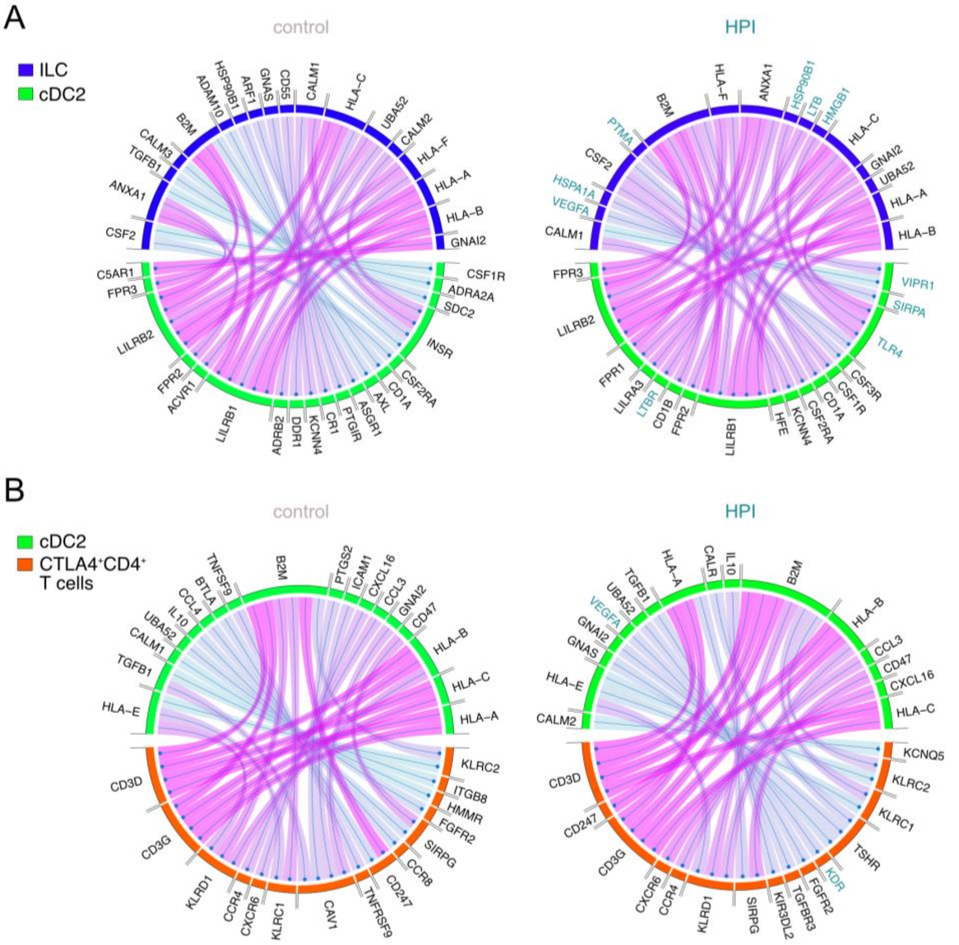

Suppl. Figure 7 | Innate and adaptive immune cells interact to form gastric TLS in the HPI tissues

a. Chord diagrams showing predicted communication pathways between ILC (ligands) and cDC2 (receptors) in HPI and uninfected tissues. **b**. Chord diagrams showing predicted communication pathways between cDC2 (ligands) and activated CD4^{+} T cells (receptors) in HPI and uninfected tissues.

Supplementary Table 1. Clinical characteristics of all patients.

**Patient ID**	**Age**	**Height**	**Weight**	**Gender**	***H. pylori* infection**	**Preoperative disease**	**Experimental purpose**			
							**FACS**	**Immuno-fluorescence**	**Microbiome ID**	**scRNA seq.**
163-11	51	167	121	Woman	Positive	Hypertension<br>Hypothyreosis	X			X
163-13	25	172.5	98	Woman	Negative	None	X			X
163-14	27	176	130	Woman	Negative	Type 2 diabetes	X			
163-15	38	161	94	Woman	Positive	None	X	X		X
163-16	64	162	90	Woman	Negative	Hypertension<br>Asthma<br>Allergy	X			
163-17	35	161	103	Woman	Negative	Thyroid disease		X		
163-22	40	189	150	Man	Negative	None	X		Control 1	X
163-24	34	160	89	Woman	Positive	None	X	X	*H.pylori* 1	
163-25	32	171	114	Woman	Negative	None	X	X	Control 2	X
163-30	32	165	104	Woman	Negative	None				
163-31	67	150	87	Woman	Positive	Hypertension<br>Atrial fibrillation	X	X	*H.pylori* 2	X
163-32	48	173	115	Woman	Negative	None		X	Control 3	X
163-33	24	164	102	Woman	Negative	Asthma				X
163-35	26	165	129	Woman	Negative	None				X

Supplementary Table 2. Antibodies used for cell sorting prior to scRNA isolation

Color	Marker	Clone	Cat. No.	Company	Used for
AlexaFluor 488 / FITC	CD3	SK7	344804	BioLegend	Lineage
	TCRα/β	IP26	306706	BioLegend	
	TCRγ/δ	B1	331208	BioLegend	
	CD94	DX22	305504	BioLegend	
	CD34	581	343504	BioLegend	
	FcεRIa	AER-37 (CRA-1)	334608	BioLegend	
PE-Cy7	CD127	R34.34	A64618	Beckman Coulter	CD127
PE-Cy5.5	HLA-DR	TU36	MHLDR18	Invitrogen	HLA-DR
APC	CD45	HI30	560973	BD Biosciences	CD45
DAPI	-	-	564907	BD Biosciences	Dead cells

Hashtag	Barcode sequence	Company	Region stained
TotalSeq™-B0251	GTCAACTCTTTAGCG	BioLegend	Fundus
TotalSeq™-B0252	TGATGGCCTATTGGG	BioLegend	Antrum

Supplementary Table 3. Antibodies used for immunophenotyping of ILCs

Color	Marker	Clone	Cat. No.	Company	Used for
AlexaFluor 488 / FITC	CD3	SK7	344804	BioLegend	Lineage + dead cells
	CD19	4G7	345776	BD Biosciences	
	CD14	TÜK4	F0844	Dako	
	CD1a	HI149	300104	BioLegend	
	CD123	6H6	306014	BioLegend	
	BDCa2	AC144	130-090-510	Miltenyi	
	TCRα/β	IP26	306706	BioLegend	
	TCRγ/δ	B1	331208	BioLegend	
	CD94	DX22	305504	BioLegend	
	CD34	581	343504	BioLegend	
	FcεRIa	AER-37 (CRA-1)	334608	BioLegend	
	Live/Dead	-	L34969	Invitrogen	
PE-Cy7	CD127	R34.34	A64618	Beckman Coulter	CD127
PE-Cy5.5	CD117	104D2D1	A66333	Beckman Coulter	CD117
PE-Cy5	Nkp44 (CD336)	Z231	A66903	Beckman Coulter	NKp44
PE-CF594	CRTH2 (CD294)	BM16	563501	BD Biosciences	CRTH2
APC	NKG2a (CD159a)	Z199	A60797	Beckman Coulter	NKG2A
QD605	CD161	HP-3G10	339916	BioLegend	CD161
AmCyan	CD45	HI30	560777	BD Biosciences	CD45

Supplementary Table 4. Antibodies used for immunofluorescence microscopy

Reagents	Clone	Cat. No.	Company
**Primary**			
Polyclonal rabbit anti-human CD3	-	GA503	Dako
Biotinylated monoclonal mouse anti-human CD20	2H7	567709	BD Biosciences
PE-conjugated monoclonal mouse anti-human CD11c	B-ly6	555392	BD Biosciences
**Secondary**			
AF488-conjugated donkey anti-rabbit antibody	-	A-21206	Invitrogen
AF647-conjugated streptavidin	-	405237	BioLegend

Supplementary Table 5. (separate excel file). DEG analysis between clusters from fundus VS antrum of figure 2.
